# From glomalin to glomalose: unraveling the molecular identity of the MAb32B11 antigen

**DOI:** 10.1111/nph.70253

**Published:** 2025-06-06

**Authors:** Burcu Alptekin, Hayley Hirsch, Bailey Kleven, Lauren King, Caitlin McLimans, Dierdra Daniels, Thomas Irving, Daniela Floss, Jean‐Michel Ané

**Affiliations:** ^1^ Department of Bacteriology University of Wisconsin – Madison Madison WI 53706 USA; ^2^ Valent BioSciences Libertyville IL 60048 USA; ^3^ Department of Plant and Agroecosystem Sciences University of Wisconsin – Madison Madison WI 53706 USA

**Keywords:** arbuscular mycorrhizal fungi, carbon sink, glomalin, HSP60, soil health

## Abstract

- Glomalin, a substance produced by arbuscular mycorrhizal (AM) fungi, has well‐documented benefits for plant and soil health, including water retention and soil aggregation. Glomalin quantification has been performed by enzyme‐linked immunosorbent assay (ELISA) using a monoclonal antibody, MAb32B11, that has been described as targeting a heat shock protein 60 (*Ri*HSP60).
- In this study, we re‐examined the molecular nature of the antigen recognized by MAb32B11.
- MAb32B11 did not cross‐react with the *Ri*HSP60 polypeptide. Glomalin extracts of *Rhizophagus irregularis* showed strong and dose‐dependent cross‐reactivity with MAb32B11 even when protein levels were undetectable, raising doubts about the proteinaceous nature of the antigen. Protease treatments of glomalin extracts did not affect the ELISA signal. However, treatment with periodate, which degrades polysaccharides, significantly reduced the signal. A strong correlation between carbohydrate content and the ELISA signal was observed in glomalin extracts.
- These findings indicate that MAb32B11 recognizes a carbohydrate, likely originating from cell walls of AM fungi. Further analysis of glomalin extracts using size exclusion chromatography suggests that the epitope of MAb32B11 is a complex carbohydrate in the size range of 511–600 kDa. Understanding the true nature of glomalin will enhance our ability to quantify it accurately and leverage its agricultural benefits.

## Introduction

Arbuscular mycorrhizal (AM) fungi belong to Glomeromycotina and form mutualistic relationships with over 70% of terrestrial plants, enhancing nutrient and water uptake and promoting plant growth (Leigh *et al*., 2009; Wang *et al*., 2017; Kakouridis *et al*., 2022). They enhance plant tolerance to biotic and abiotic stresses, including drought and heavy metals (Begum *et al*., 2019; Shi *et al*., 2023). Arbuscular mycorrhizal fungi also serve as a valuable carbon sink in the soil, storing over 10% of carbon from fossil fuel emissions, mainly as fungal necromass (Schweigert *et al*., 2015; Hawkins *et al*., 2023). This fungal necromass forms a scaffold stabilizing soil aggregates and promoting soil aggregation within mineral‐associated organic matter (Irving *et al*., 2021; Hawkins *et al*., 2023). Many of these benefits AM fungi provide are attributed to a substance they produce and release into the soil called ‘glomalin’. The abundance of glomalin in the soil has been correlated with many plant and soil health benefits, such as improved plant tolerance to abiotic stresses, increased soil aggregation, increased soil water retention, and carbon sequestration (Wright & Upadhyaya, 1996; Rillig, 2004; Zou *et al*., 2014; Zhang *et al*., 2017). This substance was discovered in 1996 by Dr. Wright after raising a monoclonal antibody named MAb32B11 against crushed *Rhizophagus irregularis* spores. The antibody was reported to be an IgM and monoclonal by Wright *et al*. (1996). The reactivity of the antibody was tested against AM fungi hyphae residing on plant roots using an immunofluorescence assay. Further efforts were also made to discover the nature of the antigen that reacts to MAb32B11 antibody, and an unusual glycoprotein was suggested to be the potential antigen for this antibody. Although glomalin was predicted to be a glycoprotein, the substance was unaffected by many harsh conditions that would degrade most proteins, such as autoclaving, sodium dodecyl sulfate (SDS), or even protease treatments (Wright *et al*., 1996; Wright & Upadhyaya, 1996).

Although always considered a protein, the definition of glomalin changed over time to reflect variations in the purification and quantification procedures used by different groups and their lack of specificity. Glomalin‐related soil protein (GRSP) was coined to reflect that many proteins besides the MAb32B11 antigen were purified in the extraction procedure. GRSP was further separated into easily extractable GRSP to define the soil proteins collected after a single autoclave cycle at 121°C with 20 mM sodium citrate pH 7.0 and total GRSP to define soil proteins collected after multiple autoclave cycles at 121°C with 50 mM sodium citrate pH 8.0 until the extract becomes ‘straw‐colored’ (Wright *et al*., 1996; Wright & Upadhyaya, 1998; Rillig, 2004; Irving *et al*., 2021). Although the addition in terminology eased confusion, it incorrectly implies that the extraction process is specific for glomalin; previous work has observed co‐extracted compounds and proteins of nonmycorrhizal origin soil glomalin extract (Nichols & Wright, 2005; Rosier *et al*., 2006; Schindler *et al*., 2007; Gillespie *et al*., 2011). The contradiction in using a nonspecific extraction process to define one unknown molecule emphasizes the knowledge gap in glomalin's molecular nature. A similar expansion in terminology was introduced to reflect nuances in glomalin quantification techniques. Two main methods have been used to quantify glomalin in the high‐temperature citrate extracts (HTCEs): enzyme‐linked immunosorbent assay (ELISA) using MAb32B11 and total protein quantification using the Bradford or Lowry methods. Initially, an indirect ELISA was designed with MAb32B11 to quantify glomalin, and this GRSP fraction was later termed immunoreactive soil proteins (IRSP; Wright *et al*., 1996; Wright & Upadhyaya, 1998; Rillig, 2004). In response to limited MAb32B11 availability, the lack of pure standard preventing absolute quantification, and the assumption of glomalin existing in the environment as an abundant soil protein, the Bradford assay followed by HTCE became a standard method for glomalin quantification in publications, and this GRSP fraction was termed Bradford‐reactive soil proteins (BRSP). However, simplicity brings additional difficulties as the Bradford assay is both nonspecific and influenced by contaminants in soil, such as lipids, humic acids, and sugars (Pande & Murthy, 1994; Nichols & Wright, 2005; Banik *et al*., 2009; Irving *et al*., 2021). The differences between the methods raise questions about their ability to equally and reliably detect glomalin.

Over the years, various protein candidates have been proposed for glomalin. Early descriptions of glomalin described it as an iron‐associated glycoprotein with N‐linked oligosaccharides similar to hydrophobins (Wright *et al*., 1996, 1998, 2000). In 2006, heat shock protein 60 (HSP60) from the AM fungus, *R. irregularis*, was proposed as the glomalin gene product based on predicted compatibility between the MAb32B11 binding sequence and the *HSP60* sequence as well as observed antibody affinity for nonglycosylated *Ri*HSP60 expressed *in vitro* (Gadkar & Rillig, 2006). However, while glomalin extract from the soil is known to be a heterogeneous mixture of compounds and proteins, *Ri*HSP60 was not detected when the glomalin extracts were analyzed with mass spectrometry (Gillespie *et al*., 2011). Similarly, in a proteomics study of *R. irregularis* mycelia, HSP60 was not detected (Murphy *et al*., 2020). In addition, the widespread conservation of the MAb32B11 epitope across fungi raised questions about the molecular nature of glomalin (Irving *et al*., 2021).

In this study, we aimed to revisit the nature of the MAb32B11 antigen and, more generally, glomalin. We showed significant discrepancies between the Bradford and ELISA techniques for quantifying glomalin. Although some fungal samples had detectable BRSPs, no IRSPs were detected. Conversely, we did not detect any BRSPs in hyphal and spore samples from AM fungi despite a strong and dose‐dependent IRSP signal. These results made us question the molecular nature of glomalin as a protein. We expressed *in vivo* and purified the *Ri*HSP60 protein, showing that MAb32B11 does not cross‐react with this protein. Furthermore, we observed a strong correlation between the amount of carbohydrates in AM extracts and the ELISA signal for glomalin. We analyzed the glomalin extract from *R. irregularis* hyphae and spores using size exclusion chromatography (SEC); we identified that the molecule is most likely a complex carbohydrate ranging in 511–600 kDa.

## Materials and Methods

### Soil sample collection

Soil samples were collected from field #B505 in April 2021 at the West Madison Agricultural Research Station (8502 Mineral Point Road, Verona, WI, USA). Maize was grown in the field the season before collection. Soil samples were stored at 4°C.

### Fungal isolates and culture conditions

*Rhizophagus irregularis* hyphae and spores, extracted by hand from chicory root and carrot root organ cultures (ROCs), were provided by Valent BioSciences (Libertyville, IL, USA) and stored at −20°C before sampling. *Rhizophagus irregularis* spores were obtained from Premier Tech (Rivière‐du‐Loup, Canada) and stored at 4°C before sampling. *Diversispora epigaea* and *Gigaspora margarita* spores were produced *in vivo* with leek and sorghum as the hosts in a 1 : 1 Quikrete Play Sand (The QUIKRETE Companies, Atlanta, GA, USA) and Turface (PROFILE Products LLC, Buffalo Grove, IL, USA) substrate mix. Spores were extracted from the substrate, collected via wet sieving, and stored at 4°C before sampling. *Mortierella elongata* NVPG4, *Gongronella butleri*, and *Mucor circinelloides* provided by the Ané Lab were grown on potato dextrose agar (PDA; Becton, Dickinson and Co., Franklin Lakes, NJ, USA) for 4–6 d at 25°C. *Umbelopsis isabellina* provided by the Ané Lab was grown on PDA for 14 d at 25°C. *Aspergillus niger* CBS 513.88, provided by Dr. Nancy Keller's Lab at the University of Wisconsin–Madison, was grown on PDA for 2–3 d at 37°C.

### Optimization of indirect ELISA protocol for glomalin quantification

The indirect ELISA protocol from Wright *et al*. (1996) was optimized to improve assay sensitivity (Supporting Information Method S1). Easily extractable glomalin was collected from soil with modification to the original protocol (Wright & Upadhyaya, 1998). The soil samples were not dried before use. The soil was homogenized by fine grinding with a blender and passing through a 500‐μm sieve. After autoclaving, samples were centrifuged for 15 min at 3000 ***g***. After glomalin extraction, total protein concentration was determined using the Bradford assay (Wright *et al*., 1996). To develop a reliable positive control for glomalin quantification, we added glomalin extract from soil in three protein concentrations. To decrease the background signal, we compared several blocking agents: 2% nonfat milk, 3% bovine serum albumin (BSA), ELISA assay buffer (5×; Thermo Scientific™, Waltham, MA, USA), and Pierce™ Protein‐Free Blocking Buffer (Thermo Scientific™). To reduce the background signal due to nonspecific binding, we compared the original secondary antibody to a secondary antibody with alkaline phosphatase conjugate (Sigma‐Aldrich, St. Louis, MO, USA).

### Heterologous expression and purification of *R. irregularis* heat shock protein 60

The coding sequence of the *R. irregularis* HSP60 (*RiHSP60*) was synthesized by Synbio Technologies (4250 US‐1 #3, Monmouth Junction, NJ, USA) with codon optimization for expression in *Escherichia coli*. The sequence was amplified and inserted into pENTR™/D‐TOPO® (Invitrogen™; Table S1). Using the Gateway™ cloning method (Thermo Scientific™), we cloned *RiHSP60* into pDEST‐His‐MBP (Addgene, Watertown, MA, USA) for expression in BL21(DE3) Competent Cells (Thermo Scientific™). Two *E. coli* strains, untransformed BL21(DE3) and BL21(DE3) with Histidine–Maltose Binding Protein (His‐MBP) expression, served as controls. Gene expression was induced with 0.5 mM isopropyl β‐d‐1‐thiogalactopyranoside (IPTG) at 30°C for 8 h; uninduced cultures were generated for comparison. Protein extraction was performed on 15 ml of culture. Cells were pelleted at 1383 ***g*** for 20 min at 4°C and then resuspended in 6 ml of extraction buffer (137 mM NaCl, 2.7 mM KCl, 10 mM Na_2_HPO_4_, 2 mM K_2_HPO_4_, and 1 mM phenylmethylsulfonyl fluoride (PMSF)). Cell suspensions were lysed with two 30‐s sonication bursts, and cell lysate was pelleted with centrifugation at 1383 ***g*** for 5 min at 4°C. The supernatant was collected for further analysis. Total protein concentration was assessed with Bradford assay (Wright *et al*., 1996. Following quantification, 15 μl of normalized protein sample, mixed with reduced 2× Laemmli Sample Buffer (Bio‐Rad, Hercules, CA, USA), was assessed with 1 mm‐thick, hand‐cast 10% polyacrylamide Tris‐Glycine sodium dodecyl‐sulfate polyacrylamide gel electrophoresis (SDS‐PAGE; Schägger, 2006). SuperSignal® Enhanced Molecular Weight Protein Ladder (Thermo Scientific™, Waltham, MA, USA) was used for comparison. Protein profiles across strains and conditions were compared after staining SDS‐PAGE gel with Coomassie Brilliant Blue R‐250 Stain Solution (Schägger, 2006). Protein expression was confirmed with modified western blot (Mahmood & Yang, 2012). Protein transfer to the polyvinylidene difluoride (PVDF) membrane was verified with Ponceau S Stain for 5 min at room temperature with agitation (1 ***g***) and destaining with deionized water. The membrane was incubated with Anti‐His antibody (Novus Biologicals, Centennial, CO, USA), diluted 1 : 2000 in blocking buffer (5% nonfat milk in Tris‐buffered saline with Tween 20 (TBS‐T) pH 7.5), for 1 h at room temperature with agitation (1 ***g***). The membrane was incubated with Goat anti‐mouse antibody IgG (H + L) (Invitrogen™, Waltham, MA, USA), conjugated to horseradish peroxidase (HRP), and diluted 1 : 20 000 in blocking solution at room temperature for 2 h with agitation (1 ***g***). Western blot was visualized with ImageQuant™ LAS 500 (GE HealthCare, Chicago, IL, USA) after treatment with Amersham™ ECL™ Prime Western Blotting Detection Reagents (Cytiva, Marlborough, MA, USA).

### Evaluation of the cross‐reactivity between RiHSP60 and MAb32B11

The ELISA was performed to assess the cross‐reactivity between MAb32B11 and *Ri*HSP60 (Method S1). Three protein concentrations, 0.25, 0.5, and 1.0 μg per well, were used for each sample with nine technical replicates. A second ELISA was conducted with an anti‐His antibody (Novus Biologicals, Centennial, CO, USA) instead of MAb32B11 (Method S1). Dot blots were performed as described previously with the following modifications (Gadkar & Rillig, 2006). *Escherichia coli* protein samples and soil glomalin extract were diluted to equal protein concentrations with 1× phosphate‐buffered saline (PBS) pH 7.4. Ten microliters of each sample was spotted on the PVDF membrane probed with MAb32B11, while 2 μl of the sample was added to the PVDF membrane probed with the Anti‐His antibody (Novus Biologicals, Centennial, CO, USA). Membranes were left to dry at room temperature for 90 min before the western blot blocking step (Mahmood & Yang, 2012). MAb32B11 was diluted 1 : 500 in blocking buffer (5% nonfat milk in TBS‐T pH 7.5), and the corresponding secondary antibody, an HRP‐goat anti‐mouse IgM (Invitrogen™), was diluted 1 : 5000 in blocking buffer. The membrane probed with the anti‐His antibody (Novus Biologicals, Centennial, CO, USA) was processed identically to the western blot. After imaging, PVDF membranes were stained with Ponceau S solution for 5 min at room temperature with agitation (1 ***g***) and destained with deionized water.

### Proteinase K treatments

To verify the insignificant effect of proteinase K treatment on glomalin, Wright *et al*. (1996) described that 6 μg of protein from soil glomalin extract was mixed with 6 μg of proteinase K (Sigma‐Aldrich, St. Louis, MO, USA) in reaction buffer (200 mM Tris‐Cl buffer pH 8.0 and 3 mM CaCl_2_). Two conditions where no proteinase K or heat‐inactivated proteinase K was added before incubation were run in parallel. The reactions were incubated for 8 h at 37°C with a heat inactivation step for 10 min at 95°C. Reactions were diluted in 1× PBS following heat inactivation to generate six technical replicates. The effect of proteinase K treatment on the cross‐reactivity between MAb32B11 and its antigen was evaluated with ELISA (Method S1).

### Periodate treatments

To validate the significant effect of periodate treatment on glomalin, Wright *et al*. (2000) described that the soil glomalin extract was concentrated with the Amicon® Ultra‐15 Centrifugal Filter (MilliporeSigma, Burlington, MA, USA). The soil sample was treated with 20 mM meta‐sodium periodate as described with modifications (Woodward *et al*., 1985). After samples had dried in wells overnight, the wells were rinsed with 250 μl of wash buffer (50 mM sodium acetate buffer pH 4.5) before 100 μl of 20 mM sodium meta‐periodate in wash buffer was added to the treated wells. The ELISA plate was incubated in the dark for 3 h at room temperature with agitation (1 ***g***). After removing periodate and washing the wells, 100 μl of 1% glycine in 1× PBS pH 7.4 was added to treated wells, and the plate was incubated at room temperature for 30 min with agitation (1 ***g***). The effect of 20 mM meta‐sodium periodate treatment on the cross‐reactivity between MAb32B11 and its antigen was evaluated with ELISA (Method S1).

### Extractions from hyphae and spores of different fungi species

Glomalin was extracted from AM fungi species, *R. irregularis*, *D. epigaea*, and *G. margarita*, hyphae, and spores with modifications (Wright *et al*., 1996). For *R. irregularis* hyphae biomass, 4–7 mg were collected, frozen with liquid nitrogen, crushed using a mortar and pestle, and mixed with 1.5 ml of 20 mM sodium citrate buffer pH 7.0. Increasing amounts of *R. irregularis* spore biomass (5, 10, 25, 50, 75, 100, and 250 mg) were similarly frozen and crushed and then mixed with 2 ml of 20 mM sodium citrate buffer pH 7.0. All samples were autoclaved for 30 min at 121°C. After autoclaving, samples were centrifuged at 3000 ***g*** for 15 min, and the supernatant was collected for further analysis. Total protein concentration in glomalin extracts was determined using the Bradford assay (Wright *et al*., 1996). To evaluate the glomalin signal from *R. irregularis* hyphae extract, 50 μl of pure glomalin extract was added to ELISA wells (Method S1). Total carbohydrate concentration within glomalin extracts from *R. irregularis* spores was assessed with the Total Carbohydrate Assay Kit (Sigma‐Aldrich), and absorbance was measured at 450 nm (Nielsen, 2017). To evaluate the glomalin signal from *R. irregularis* spore extract, the glomalin extract generated from 25 mg of *R. irregularis* spore biomass was diluted to 0.05, 0.5, and 5 μg of carbohydrate per ELISA well (Method S1). Similarly, glomalin extracts with 30–50 mg ml^−1^ spore biomass concentrations were generated from *D. epigaea* and *G. margarita* and evaluated with ELISA (Method S1). Glomalin signal from *Aspergillus niger* (Ascomycota), *Gongronella butleri* (Zygomycota), *Mortierella elongata* (Mucoromycota), *Mucor circinelloides* (Mucoromycota), and *Umbelopsis isabellina* (Mucoromycota) hyphae/spore samples were evaluated similarly to AM fungi. After growing the fungus in appropriate conditions and media, hyphae/spore biomass (4–7 mg) were collected, frozen with liquid nitrogen, crushed using a mortar and pestle, and mixed with 1.5 ml of 20 mM sodium citrate buffer pH 7.0. As explained above, these samples were processed through glomalin extraction, Bradford, and ELISA procedures.

### Size exclusion chromatography and fractionation of glomalin extract

Glomalin extract from *R. irregularis* spores and hyphae was analyzed with SEC and fractionation at the Complex Carbohydrate Research Center at the University of Georgia. Glomalin from *R. irregularis* spores and hyphae was extracted as described in Method S1 with *c*. 5 g of fungal hyphae and spores, using 50 mg of fungal material per ml of sodium citrate (20 mM). Following the extraction procedure, the carbohydrate amount in the extract was quantified using the Total Carbohydrate Assay Kit (Sigma‐Aldrich). Approximately 35 mg of fungal carbohydrates were fractionated and analyzed by the SEC system on a Superose 6 column (10 × 300 mm, Cytiva) with a refractive index (RI) detector. A series of dextran standards (0.5 mg ml^−1^) with peak average molecular weights between 6 and 500 kDa was used to calibrate the Superose 6 column. Twenty fractionations were performed, collecting six fractions each time. Fractions were then returned to the University of Wisconsin–Madison to test their reactivity with the MAb32B11 antibody with ELISA. After testing different fractions with ELISA using the MAb32B11 antibody, Fraction 2 was chosen for further analysis with 1D Nuclear Magnetic Resonance (NMR) spectroscopy to detect the glycosyl composition of the fragment and with 2D NMR to determine the structure and the sequence of the carbohydrate epitope.

### Testing different fungal cell wall components as a glomalin candidate

Different carbohydrates commonly residing in fungal cell walls were tested for cross‐reactivity with MAb32B11. As a potential candidate for β‐glucans, laminarin and its derivatives laminaritetraose, laminaripentose, and laminarihexose (all were obtained from Megaenzyme, LTD) were prepared by dissolving the chemical in water at 0.01 M concentration. Chitin oligomers tetra‐N‐acetyl‐chitotetraose (CO_4_) and hepta‐N‐acetyl‐chitoheptaose (CO_7_) were prepared in‐house and dissolved in 50% EtOH at 10–5 M concentration. Amylose, amylopectin, and glycogen (obtained from Millipore Sigma, Burlington, MA, USA) were dissolved in hot water at 5 M concentration.

### Statistical analyses

Statistical data analyses were performed using the R software (v.4.2.3). Normality across data sets was evaluated with the Shapiro–Wilk normality test. Significant differences in ELISA signal across four blocking agents (*n* = 12) were assessed with one‐way ANOVA with *post hoc* Tukey's honest significant difference test. The ELISA signals from compared secondary antibodies (*n* = 12) were evaluated with unpaired, two‐tailed Student's *t*‐test. Graphic illustrations were generated with R package ggplot2 (R v.4.2.2), and the significance level was set to *, *P* ≤ 0.05; **, *P* ≤ 0.01; ***, *P* ≤ 0.001. All raw data related to ELISA signals are provided in Dataset S1.

## Results

### Indirect ELISA with MAb32B11, but not Bradford assay, offers a reliable quantification of glomalin

**Fig. 1:**
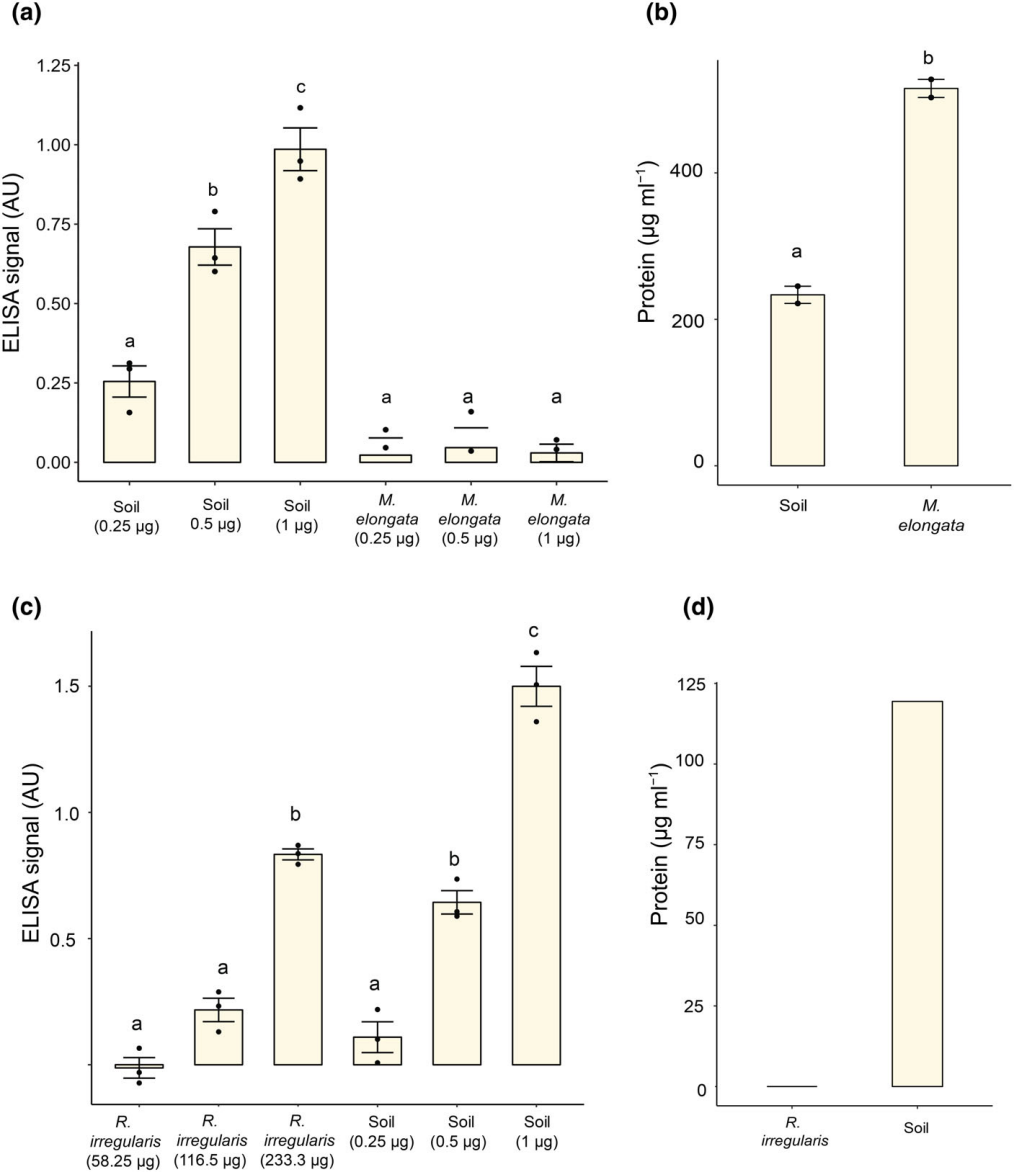
Discrepancy between enzyme‐linked immunosorbent assay (ELISA) and Bradford data for glomalin quantification. Glomalin signal in arbitrary units (AU) based on indirect ELISA with MAb32B11 using soil (a, c), *Mortierella elongata* (b), and *R. irregularis* hyphae/spore samples (c). Soil samples show an increased amount of glomalin signal in response to an increased amount of antigen, which was provided by increasing the amount of soil proteins (a, c); however, *M. elongata* samples did not show this signal increase (one‐way ANOVA, *n* = 3, *P* < 0.05) (a). *R. irregularis* hyphae/spore samples showed an increased amount of glomalin signal in response to an increased amount of antigen, which was provided by increasing the biomass of hyphae/spore samples (one‐way ANOVA, *n* = 3, *P* < 0.05) (c). Bradford‐reactive proteins from soil (b, d), *M. elongata* (b), and *R. irregularis* hyphae/spore samples (d) after the hot citrate buffer extraction process. No protein was detected in *R. irregularis* hyphae/spore samples after the hot citrate buffer extraction process (d). Statistical significances on (b) are based on one‐way ANOVA *post hoc* Tukey's honest significant difference test (*n* = 2, *P* < 0.05). Letters above the bars indicate statistically significant differences between samples. Error bars represent the SE of the mean.

We first aimed to compare the ELISA with MAb32B11 and Bradford quantification methods for glomalin. We used soil collected from the West Madison Agricultural Station (WMARS) as a positive control. We compared it to fungal biomass from *Mortierella elongata* NVP64, a fungus belonging to the Mucoromycota phylum like AM fungi but not mycorrhizal. We normalized the ELISA data based on protein levels as described by Wright *et al*. (1996). We observed an increased ELISA signal with increased amounts of soil proteins (Fig. 1a). A minimal ELISA signal was detected with *M. elongata* extracts despite containing 0.25–1 μg of BRSPs (Fig. 1a,b). More importantly, the weak signal did not vary with increasing amounts of *M. elongata* proteins, thus highlighting the importance of checking the dose dependence of this assay to increased amounts of epitopes to ensure specificity (Fig. 1a). Interestingly, based on the Bradford assay, our *M. elongata* extract had more BRSP than the soil sample. Still, no IRSP was detected in this extract, highlighting the total lack of specificity of the BRSP approach. Results similar to those presented with *M. elongata* were obtained with the ascomycete *Aspergillus niger*, the zygomycete *Gongronella butleri*, and two additional Mucoromycota fungi (*Mucor circinelloides* and *Umbelopsis isabellina*; Fig. S1). Therefore, we focused our study on the ELISA with MAb32B11 and will refer to the MAb32B11 epitope as ‘glomalin’.

### Optimization of the ELISA with MAb32B11 to quantify glomalin

We noticed a high background in ELISA assays with even blank (PBS buffer) samples using the original procedure (Wright *et al*., 1996). Given the importance of this ELISA with MAb32B1 for glomalin quantification, we aimed to improve this protocol and reduce the background signal. A summary of changes is provided in Fig. S2a. We tested three commonly used blocking buffers, Invitrogen™ Blocking Buffer, Pierce™ Blocking Buffer, and 3% BSA, to decrease the potential role of the blocking step on this issue. As shown in Fig. S2b, 2% milk provided the lowest background signal; therefore, it was kept as the blocking buffer (Wright *et al*., 1996). The 1996 protocol suggested using a goat anti‐mouse antibody bound to biotin, which can be detected by incubating alkaline phosphatase bound to streptavidin (Wright *et al*., 1996). As shown in Fig. S2c, this additional step of streptavidin incubation increases the background signal in the indirect ELISA. By changing this antibody with a goat anti‐mouse alkaline phosphatase‐conjugated IgG, the background signal was reduced, and the sensitivity of the indirect ELISA was significantly improved (Fig. S2c). Furthermore, this change in the protocol reduced the assay time to *c*. 1 h. We also optimized glomalin signal detection at the p‐nitrophenyl phosphate (pNPP) incubation step. Glomalin signal from field soil samples with three different BRSP concentrations (0.25, 0.5, and 1 μg) was assessed after 15, 30, 45, and 60‐min incubation with pNPP. Fig. S2d shows that the glomalin signal can be well detected after 45 min incubation with pNPP. For the convenience of sample storage and ease of processing, it is standard practice to air‐dry soil. The standard procedure to quantify glomalin in soil samples is to air‐dry the soil sample (Wright & Upadhyaya, 1998). We tested the effect of soil air drying on the ELISA signal. We showed that increased drying leads to decreased ELISA signal, indicating that soil air drying significantly impacts ELISA data and should be consistent across samples for accurate comparisons (Fig. S3). Therefore, shorter air‐drying times are recommended for maximal sensitivity (Fig. S3).

### The epitope recognized by MAb32B11 is not the heat shock protein 60 of AM fungi

**Fig. 2:**
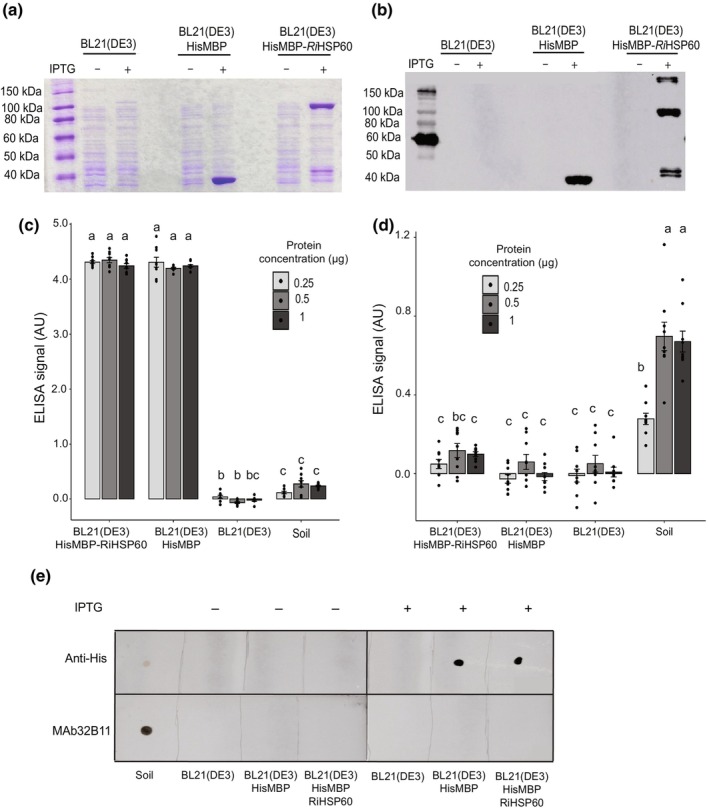
*Rhizophagus irregularis* heat shock protein 60 (HSP60) production and absence of cross‐reactivity with MAb32B11. (a, b) Sodium dodecyl sulfate polyacrylamide gel electrophoresis (SDS‐PAGE) and anti‐His western blot showing supernatant protein profiles from induced (IPTG+) and not induced (IPTG−) conditions with not transformed *Escherichia coli* supernatant (BL21(DE3)), *E. coli* transformed with HisMBP tag (BL21(DE3) HisMBP), and *E. coli* transformed with HisMBP tagged *RiHSP60* BL21(DE3) HisMBP‐BL21(DE3). (c) Anti‐His enzyme‐linked immunosorbent assay (ELISA) with *E. coli* supernatant from IPTG + condition. (d) MAb32B11 ELISA with *E. coli* supernatant from IPTG+ condition. (c, d). Dots represent technical replicates, and bars represent mean ELISA signal ± SEM (Kruskal–Wallis with *post hoc* pairwise Wilcoxon rank sum test, *n* = 9, *P* < 0.05). (c, d) Legend indicates micrograms of protein added to ELISA wells (0.25, 0.5, and 1 μg) and y‐axis unit, arbitrary units (AU), represents ELISA signal normalized to absorbance at 405 nm (A_405_) in wells containing only 1× PBS (blank wells). (e) MAb32B11 dot blot compared with anti‐His dot blot using *E. coli* supernatant collected from induced (IPTG+) and not induced (IPTG−) conditions. (c–e) Easily extractable glomalin extract collected from soil (Soil) was used as a positive control for glomalin detection. (c, d) Significant differences in ELISA signal across *E. coli* protein samples and soil glomalin extract were determined with the Kruskal–Wallis and *post hoc* pairwise Wilcoxon rank sum tests. Letters above the bars indicate statistically significant differences between samples. Error bars represent the SE of the mean.

With the newly optimized ELISA, we first tried to use purified *Ri*HSP60 as a reference for absolute quantification of glomalin. We expressed *Ri*HSP60 with a His‐MBP tag in E*scherichia coli* and evaluated its cross‐reactivity with MAb32B11 (Fig. 2a,b). We assessed the cross‐reactivity of HisMBP::*Ri*HSP60 with MAb32B11 by ELISA and dot blot. To our surprise, although HisMBP‐tagged *Ri*HSP60 was present and gave reactivity with the anti‐His antibody, we did not observe any cross‐reactivity with MAb32B11 based on indirect ELISA (Fig. 2c,d). Gadkar & Rillig (2006) used dot blots for similar experiments with MAb32B11 but, in our case, using a 1/500 dilution of MAb32B11, no cross‐reactivity between MAb32B11 and HisMBP::*Ri*HSP60 was observed (Fig. 2e). Together, our results indicate that the glomalin antibody, MAb32B11, does not recognize the *Ri*HSP60 polypeptide.

### The epitope recognized by MAb32B11 is not even a protein

**Fig. 3:**
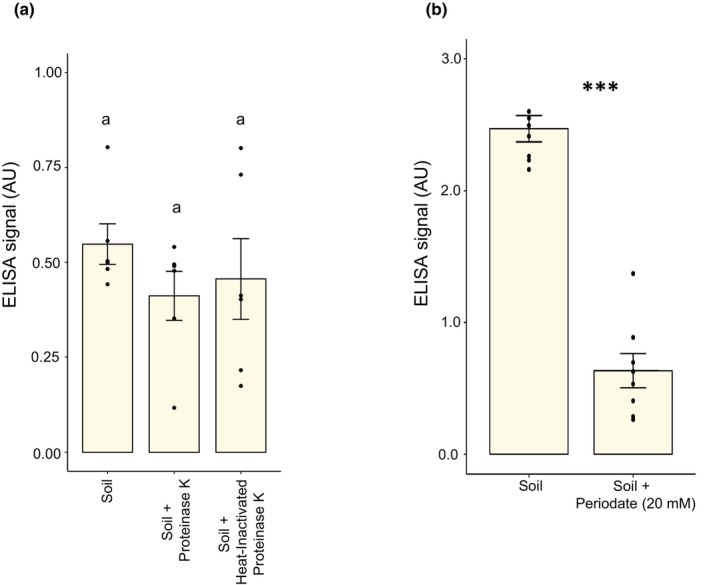
Enzyme‐linked immunosorbent assay (ELISA)‐based glomalin signal does not originate from a protein. (a) Proteinase K treatment of glomalin extracts from soil samples is shown. Glomalin extracts from field soil were treated with proteinase K enzyme, a broad‐spectrum serine protease, and indirect ELISA with MAb32B11 was performed to assess the change in the glomalin signal in response to the degradation of proteins. The proteinase K treatment did not significantly affect the ELISA‐based glomalin signal (one‐way ANOVA with *post hoc* Tukey's honest significant difference test, *n* = 6, *P* < 0.05). (b) The impact of Periodate treatment on the reactivity of MAb32B11 with unknown glomalin antigen is shown. Concentrated glomalin extracts from field soil were treated with 20 mM periodate. This chemical leads to cleavage of carbohydrate vicinal hydroxyl groups, and indirect ELISA with MAb32B11 was performed to assess the change in the glomalin signal (in arbitrary unit (AU)). The Periodate treatment significantly affected the reactivity of the glomalin antigen with MAb32B11, leading to the loss of > 70% of reactivity (Wilcoxon test, *n* = 8, *P* < 0.001 represented with ***). Letters above the bars in (a) indicate statistically significant differences between samples. Error bars represent the SE of the mean.

We compared hot citrate extracts from WMARS field soil to those from *R. irregularis* (spores and hyphae) grown *in vitro* from carrot and chicory ROCs. As expected, the ELISA signal presented a dose‐dependent response to fungal biomass amount, but, to our surprise, we could not detect any proteins in these extracts, preventing us from normalizing these ELISA data to protein levels (Fig. 1c,d). Previous work (Wright *et al*., 1996) suggested that the antigen for MAb32B11 is resistant to proteases (pronase, trypsin, and endoglycosidase‐H assessed by immunofluorescence), but unfortunately, these data were not shown. We performed this assay by treating glomalin extracts from field soil samples with proteinase K, a broad‐spectrum serine protease. Before this analysis, we tested the activity of proteinase K using BSA (Fig. S4). We observed no difference between proteinase K‐treated and nontreated samples in the ELISA‐based glomalin signal (Fig. 3a).

### The epitope recognized by MAb32B11 is a complex carbohydrate

Previous work suggested that glomalin is a glycoprotein based on the presence of cleaved carbohydrates on high‐performance capillary electrophoresis analysis (Wright *et al*., 1996). These authors also reported that periodate treatment eliminates the immunofluorescence of glomalin on hyphae, but again, unfortunately, no data or periodate concentration was shown. Periodate oxidizes polysaccharide chains, producing aldehyde groups (Bobbitt, 1956; Woodward *et al*., 1985). We treated glomalin extracts from field soil samples with periodate to test the possibility of the MAb32B11 antigen being a carbohydrate rather than a protein. Even a mild (20 mM) periodate treatment reduced the reactivity of the antigen with MAb32B11 by over 60% (Fig. 3b). This result strengthened our hypothesis of glomalin being a carbohydrate rather than a protein.

**Fig. 4:**
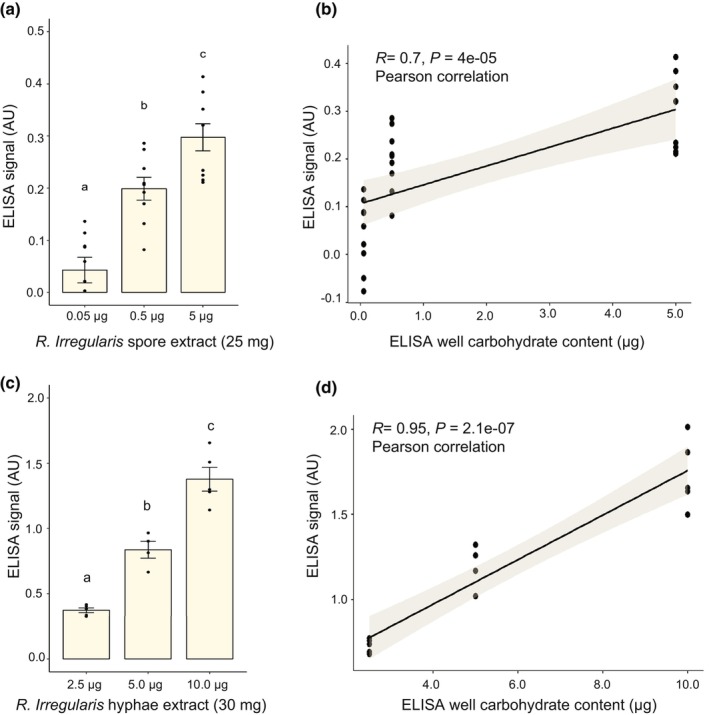
MAb32B11 antigen abundance correlates with the total carbohydrate concentration in arbuscular mycorrhizal (AM) spores and hyphae. (a, b) A significant positive correlation was observed between the carbohydrate amount added to per well (*n* = 9) and the enzyme‐linked immunosorbent assay (ELISA)‐based glomalin signal from the glomalin extracts of *R. irregularis* spores. Reactivity of the glomalin antigen with MAb32B11 was elevated in response to increased concentration of carbohydrates per well, indicating the enrichment of the glomalin antigen by adding more carbohydrates (one‐way ANOVA, n = 9, *P* < 0.05). (c, d) A similar correlation between the carbohydrate amount added per well (*n* = 5) and the ELISA‐based glomalin signal was also observed for glomalin extracts of *R. irregularis* hyphae (one‐way ANOVA, *P* < 0.05). No protein was detected in glomalin extracts of *R. irregularis* hyphae and spore samples. Differences in ELISA signal across ELISA wells containing varying amounts of glomalin extract from *R. irregularis* spores and hyphae were determined with one‐way ANOVA with *post hoc* Tukey's honest significant difference test. Pearson correlation was used to determine the relationship between the ELISA signal and the amount of carbohydrate from *R. irregularis* glomalin extract. The shaded area around the regression line in (b) and (d) represents the 95% confidence interval. Letters above the bars indicate statistically significant differences between samples. Error bars represent the SE of the mean.

We noticed that the hot citrate extraction method developed by Wright *et al*. (1996) looks strikingly similar to standard protocols used to extract fungal polysaccharides (Peat *et al*., 1961; Kocourek & Ballou, 1969; Nakajima & Ballou, 1974). Fig. S5 shows that increasing the amount of *R. irregularis* spores used in the glomalin extraction process leads to increased total carbohydrates in the extract. A strong positive correlation (*R* = 0.96, *P* = 2^−12^, Pearson correlation) was observed between the amount of spore biomass and carbohydrates. Again, no protein was detected in any of these spore samples, like what we observed with *R. irregularis* hyphae and spores (Figs 1, S5). We hypothesized that, if the antigen for MAb32B11 is a polysaccharide, there should be an increase in the indirect ELISA‐based glomalin signal in response to the increased concentration of total carbohydrates. Glomalin extracts from *R. irregularis* spores and hyphae, collected from chicory ROCs, were used to test this hypothesis. Although no protein was again detected in glomalin extracts from both *R. irregularis* spores and hyphae samples, we observed a strong glomalin signal from both samples (Fig. 4). Furthermore, we observed a substantial increase in the glomalin signal in response to increased concentration of carbohydrates per well in the indirect ELISA assay (Fig. 4a,b). We also observed a strong correlation between the carbohydrate concentration and the ELISA signal (*R* = 0.7, *P* < 0.001). A similar correlation was also observed for glomalin extracts from spore samples (Fig. 4c,d; *R* = 0.95, *P* < 0.001).

**Fig. 5:**
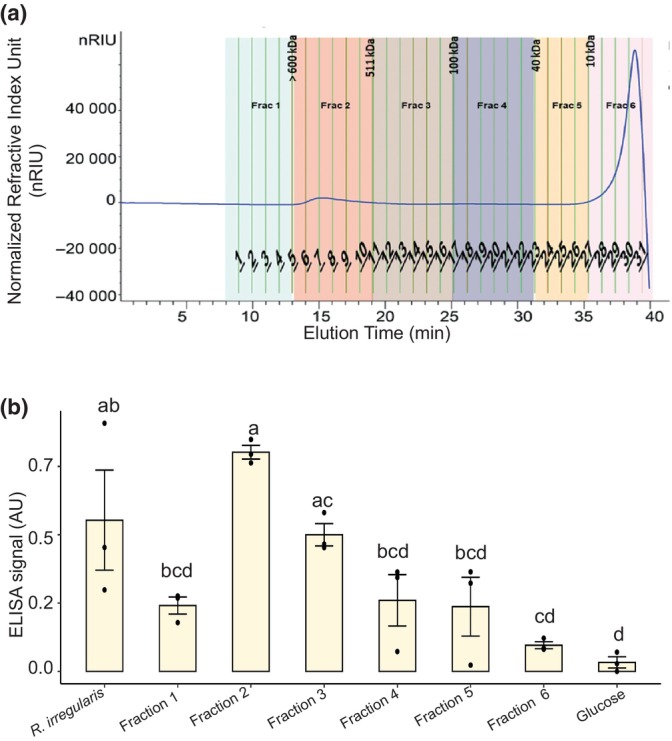
Size exclusion chromatography (SEC) of glomalin extract from *Rhizophagus irregularis* spore samples. The *R. irregularis* glomalin extract was fractionated and analyzed by SEC on a Superose 6 column. The SEC refractive index signal profile of fractions is represented in (a). Fractions were then tested for cross‐reactivity with the MAb32B11 antibody using 50 μl of fraction before carbohydrate quantification. Fractions 2 and 3 showed a significant signal (in arbitrary unit (AU) in the enzyme‐linked immunosorbent assay (ELISA) (one‐way ANOVA with *post hoc* Tukey's honest significant difference test, *P* < 0.05). (b) To identify the fraction with a true glomalin signal, the carbohydrate content of each fraction was quantified and ELISA was repeated with 7.5 μg of carbohydrates per ELISA well. Letters above the bars in (b) indicate statistically significant differences between samples. Error bars represent the SE of the mean.

To explore the possibility that MAb32B11 binds to a known polysaccharide in the cell wall of AM fungi, we tested a few target polysaccharides commonly found in fungal cell walls using commercially available molecules. Selected oligomers of chitin (CO_4_ and CO_7_), α‐glucans (amylose, amylopectin, and glycogen), and β‐glucans (laminarin and its derivatives). Unfortunately, none showed any reactivity with MAb32B11 (Fig. S6). Due to the failure of our candidate approach, we turned to a combination of SEC, GC‐MS, and NMR. The *R. irregularis* glomalin extract from spore samples was analyzed by SEC with the Complex Carbohydrate Research Center at the University of Georgia. The extract was separated into six fractions ranging from 5 to 700 kDa. Fractions were tested for reactivity with the MAb32B11 antibody (Fig. 5a). Due to the low amount of sample, we were not able to quantify the carbohydrate content in each fraction and directly tested fractions with ELISA using 50 μl of sample in three technical replicates. Based on ELISA with the MAb32B11 antibody, both Fraction 2 (600–511 kDa) and Fraction 3 (500–100 kDa) showed a robust signal with ELISA (Fig. 5b). Since the signal coming from Fraction 2 was the highest, we continued with this fraction for further analysis. Further analysis of Fraction 2 by trimethylsilyl derivatization and GC‐MS revealed the presence of rhamnose (Rha), glucose (Glc), arabinose (Ara), fucose (Fuc), galactose (Gal), mannose (Man), glucuronic acid (GlcA), and N‐acetylgalactosamine (GalNAc) in this fraction (Table S2). The composition of Fraction 2 was further analyzed using 1D and 2D NMR. This analysis revealed that Fraction 2 contains mostly a linear polysaccharide with tetra‐saccharide repeating units. This structure has a Rha (residue A) is α‐(1 → 3) linked to a Glc (residue D), which is β‐(1 → 4) linked to a GlcA (residue C) that forms a β‐(1 → 4) link with a Glc (residue B), which connects via a β‐(1 → 4) linkage back to residue A (Fig. S7a). To our surprise, this structure is the same as gellan gum produced by *Sphingomonas elodea* and classically used for *in vitro* culture of AM fungi (Jay *et al*., 1998; Cheng & Neiss, 2012; Ghorui *et al*., 2023). We tested gellan gum's capacity to bind the MAb32B11 antibody and, as expected, gellan gum is not bound by the MAb32B11 antibody (Fig. S7b). Therefore, we conclude that the antigen of the MAb32B11 antibody is most likely a polysaccharide in Fraction 2. However, the abundance of gellan gum in these extracts from AM fungi grown *in vitro* prevented us from identifying its structure.

### The epitope recognized by MAb32B11 is quite specific to AM fungi

**Fig. 6:**
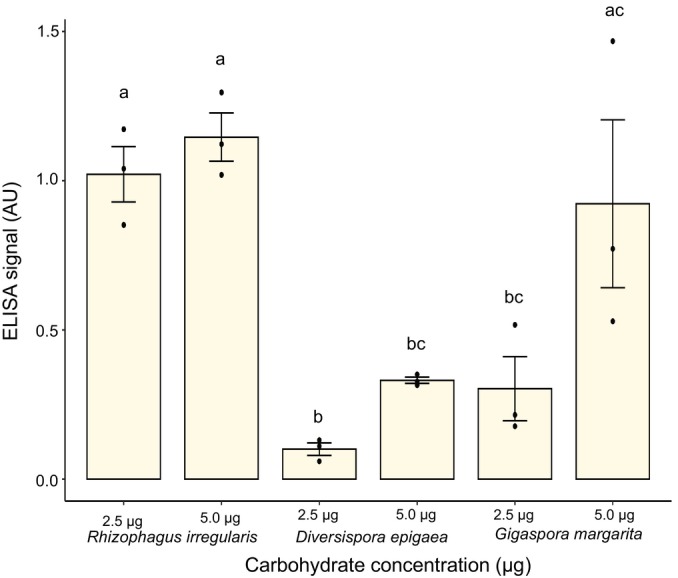
Abundance of the MAb32B11 antigen in various species of arbuscular mycorrhizal (AM) fungi. *Diversispora epigaea* and *Gigaspora margarita* spore sample*s* showed the glomalin signal (in arbitrary unit (AU)) based on indirect enzyme‐linked immunosorbent assay (ELISA) performed using MAb32B11. There was a statistically significant effect of carbohydrate concentration on the indirect ELISA‐based glomalin signal (two‐way ANOVA, *n* = 3, *P*‐value = 0.02); however, individual comparisons did not have significance (Tukey's honest significant difference test, *P*‐value > 0.05). Letters above the bars indicate statistically significant differences between samples. Error bars in the graph represent SE of the mean.

Glomalin extractions from *R. irregularis in vitro* culture led to undetectable levels of proteins, making normalization to protein levels impossible. In addition, given our previous data showing that the MAb32B11 antigen is a polysaccharide rather than a protein, it makes sense to normalize the ELISA data by total carbohydrate content rather than protein levels. In addition to *R. irregularis*, we quantified glomalin in spores for two other species of mycorrhizal fungi, *Gigaspora margarita* and *Diversispora epigaea*, grown *in vitro*. No protein was detected in the *G. margarita* spore extract, while only 0.044 μg μl^−1^ protein was observed in *D. epigaea* one. Both samples contained a substantial amount of carbohydrates (*D. epigaea*: 0.121 μg μl^−1^ and *G. margarita*: 0.245 μg μl^−1^ of total carbohydrates). Indirect ELISA with MAb32B11 showed that glomalin extracts from *G. margarita* and *D. epigaea* spore samples have a substantial glomalin signal (Fig. 6). We also quantified glomalin in *in vitro* cultures of nonmycorrhizal fungi *Mortierella elongata* and *Mucor circinelloides*. Hyphae and spore samples of *M. elongata* and *M. circinelloides* did not show any glomalin signal despite having a substantial amount of carbohydrates (*M. elongata*: 0.137 μg μl^−1^ and *M. circinelloides*: 0.180 μg μl^−1^ of total carbohydrates; Fig. S1). Therefore, our results suggest that MAb32B11 reacts quite specifically with polysaccharides in AM species.

## Discussion

The term ‘glomalin’ was first introduced into the scientific literature over 25 yr ago with Dr. Sarah Wright's description of an abundant protein produced by AM fungi (Wright *et al*., 1996). Since its discovery, Glomalin has been associated with many soil health benefits, including soil aggregation and soil water retention (Rillig, 2004; Jansa *et al*., 2020; Holátko *et al*., 2021; Singh *et al*., 2022). Despite the report that MAb32B11 binds an HSP60 from *R. irregularis*, the nature of glomalin and GRSP remains controversial (Gadkar & Rillig, 2006;Gillespie *et al*., 2011; Irving *et al*., 2021). In this work, we attempted to clarify the molecular nature of glomalin by revisiting the nature of the MAb32B11 epitope.

### Traditional approaches to quantifying glomalin need to be revised

Glomalin was initially quantified using the Bradford assay and ELISA with a monoclonal antibody, MAb32B11, raised against crushed AM fungal spores (Wright *et al*., 1996). However, later research mainly used the Bradford assay to quantify glomalin due to the assumption that glomalin was a protein and the limited availability of MAb32B11 (Irving *et al*., 2021). Although it was shown that glomalin extracts contain other compounds and heat‐stable proteins of nonmycorrhizal origins, the Bradford assay has been the primary way of quantifying glomalin for many years (Rosier *et al*., 2006; Gillespie *et al*., 2011). Our study showed that hot citrate extracts from *M. elongata* biomass contained more Bradford‐reactive protein than the soil samples, but unlike the soil samples did not show reactivity in MAb32B11 ELISA (Fig. 1). Reciprocally, glomalin extracts from spores and hyphae showed good ELISA responses but contained no detectable protein levels using the Bradford assay which is sensitive down to 50 ng of BSA protein (Bradford, 1976; Ernst & Zor, 2010). The Bradford assay on the *M. elongata* biomass likely quantified heat‐stable proteins that did not precipitate through the autoclaving process in citrate buffer. Our results corroborate previous findings and indicate that using the Bradford assay is misleading for glomalin quantification (Gillespie *et al*., 2011; Irving *et al*., 2021). Indirect ELISA using MAb32B11 should be used as a standard for quantifying glomalin. At the same time, ELISA's reliability and sensitivity depend on the antibody and its characteristics, such as epitope specificity, purity, and affinity (Schmidt *et al*., 2005). Since the complete identity of the antigen for MAb32B11 antibody is still elusive, extra precautions should be taken into consideration to ensure ELISA is specific to the molecule of interest. Using the dose dependency of the ELISA response should be standard procedure. For instance, different amounts of soil or fungal extract should show an increase in ELISA signal with increased amounts of starting material (Fig. 1). The high nonspecific background signal in MAb32B11 ELISA can decrease sensitivity. We tested the effect of the blocking reagent, secondary antibody, and incubation time in pNPP. The significant improvement in reducing ELISA background was replacing the goat anti‐mouse antibody bound to biotin with one conjugated with the alkaline phosphatase (Fig. S2). We also propose to stop using arbitrary soil samples as a reference to calculate an artificial amount of glomalin based on ELISA data. There is no standard for glomalin, so these calculations are misleading. Until we purify the MAb32B11 antigen, we propose that the community use the ELISA assays as a relative quantification measure rather than an absolute one and report the data in arbitrary units. As unsatisfying as this can be, this would clarify our knowledge of glomalin. Identification of the MAb32B11 actual antigen will be necessary for the absolute quantification of glomalin.

### The RiHSP60 protein is not glomalin

Revealing the molecular nature of glomalin is essential to fully understand its contributions to soil and plant health. In 2006, Gadkar & Rillig took a step in this direction by partially sequencing a protein that seemed to show reactivity with MAb32B11 (Gadkar & Rillig, 2006). This work suggested *R. irregularis* HSP60 (*Ri*HSP60) as a candidate for glomalin. However, as discussed very clearly by the authors, it is difficult to reconcile this hypothesis with many properties of glomalin (Gillespie *et al*., 2011; Irving *et al*., 2021). For instance, HSP60 is a highly conserved protein in the fungal domain, which does not match the idea of an AM fungi‐specific protein, as glomalin was suggested (Irving *et al*., 2021). It is also difficult to imagine how *Ri*HSP60 could confer benefits, such as soil aggregation or water retention (Irving *et al*., 2021). With the initial aim of developing a standard for absolute quantification by ELISA and considering these inconsistencies, we decided to revisit whether *Ri*HSP60 is a strong candidate for glomalin. Unfortunately, our results failed to support the hypothesis that MAb32B11 recognizes the *Ri*HSP60 polypeptide (Fig. 2). It is possible that the previous authors observed unspecific binding between *Ri*HSP60 and MAb32B11 since they used a high antibody concentration in their dot blot assay (1 : 2). By contrast, we used a much more conventional antibody dilution (1 : 500; Gadkar & Rillig, 2006). In addition, we used ELISA to confirm the dot blot results (Fig. 2). Our results indicate that the *Ri*HSP60 polypeptide is likely not the MAb32B11 antigen.

### From ‘glomalin’ to ‘glomalose’

Glomalin was described as a proteinaceous molecule for > 25 yr (Wright *et al*., 1996). Hence, its characterization and quantification were carried out with the assumption of its proteinaceous nature. However, Wright's glomalin extraction method does not specifically extract proteins. Furthermore, Wright's own work described the antigen of MAb32B11 as an unusually tough molecule resistant to treatments like extreme heat, pH, SDS, and protease treatments that disrupt protein integrity (Wright *et al*., 1996). Here, we observed that an antigen from glomalin extracts of *R. irregularis* reacts with MAb32B11, despite the lack of proteins in the extracts, quantified using the Bradford assay (Fig. 1). Since the Bradford assay is a highly sensitive technique to quantify proteins (Bradford, 1976), this result is hard to reconcile with glomalin being an abundant protein produced by AM fungi. Glomalin was reported as resistant to proteases (pronase, trypsin, and endoglycosidase‐H as assessed by immunofluorescence) but, unfortunately, without showing any data (Wright *et al*., 1996). Our experiments confirmed that the MAb32B11 antigen is insensitive to proteinase K (Fig. 3a), which seems surprising for a protein. It was also reported that the glomalin signal by *in situ* hybridization is sensitive to periodate, but again, no data were shown (Wright *et al*., 2000). Our ELISA data (Fig. 3b) confirmed this observation, suggesting that the MAb32B11 antigen is a polysaccharide.

An increase in carbohydrate concentration led to an enrichment of the antigen for MAb32B11 (Fig. 4). The absence of proteins from glomalin extracts of hyphae and spore samples of *R. irregularis* further corroborates the nonproteinaceous nature of the MAb32B11 antigen. Besides, some carbohydrate extractions are strikingly similar to the glomalin extraction method (Peat *et al*., 1961; Nakajima & Ballou, 1974). Mannans and mannan oligosaccharides from many fungi can be extracted by autoclaving samples for 90 min in 0.02 M of citrate buffer (Kocourek & Ballou, 1969). Mannans are freely soluble in water and can be extracted by only disrupting the cells with autoclaving (Peat *et al*., 1961). Fig. S5 shows that the glomalin extraction process from *R. irregularis* spores extracts more carbohydrates in response to increased spore biomass. No protein was detected from the spore samples used in this assay (Fig. 4b). Altogether, our data indicate that the glomalin extraction process extracts a polysaccharide and not a protein recognized by MAb32B11. To represent its carbohydrate nature, we propose a new name for the MAb32B11 antigen: glomalose. It is possible that the target of MAb32B11 may be a family of compounds. We attempted to identify the glomalose using SEC, GC‐MS, and NMR. This analysis took over a year due to the amount of fungal material needed (*c*. 5 g of fungal spore and hyphae). Fractioning the glomalose extract and testing the reactivity of fractions with the MAb32B11 antibody suggested that glomalose is a molecule in the size range of 600–511 kDa. This result suggests that glomalose is a long polysaccharide. Unfortunately, neither our candidate approach nor the biochemical purification allowed us to identify the polysaccharide in the present study. The only polysaccharide we were able to identify by NMR in Fraction 2 was gellan gum. Gellan gum is the primary gelling agent used in *in vitro* cultures for growing mycorrhizal fungi (Fernández Bidondo *et al*., 2016; Ghorui *et al*., 2023). As demonstrated in Fig. S7, gellan gum itself does not react with the MAb32B11 antibody. We suspect that the glomalose molecule is within this Fraction 2, but it was not detected by NMR due to its lower abundance than gellan gum. Further strategies must be developed to grow AM fungal spores and hyphae without gellan gum to purify glomalose for structural analysis.

### Working model for glomalose and its multiple benefits

**Fig. 7:**
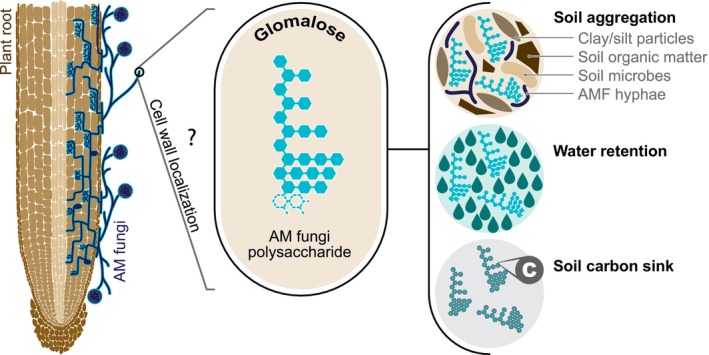
From glomalin to glomalose: a paradigm shift for the molecular identity of the MAb32B11 antigen. We hypothesize that glomalose is an abundant polysaccharide specific to arbuscular mycorrhizal (AM) fungi and resides in fungal hyphae and spores. Glomalose is most likely a part of mycorrhizal fungal cell walls and is released in the soil during fungal turnover. After its release into the soil, it performs essential functions attributed to glomalin. Glomalose assists in binding soil organic matter and other soil particulates, such as clay and silt, and helps with soil aggregation. Glomalose can also contribute to soil aggregation by serving as a food source for other microorganisms. Glomalose is hypothesized to be a hydrophilic water‐soluble polysaccharide, possibly helping with water retention in the soil. Furthermore, glomalose is a valuable soil carbon sink due to its highly stable polysaccharide nature.

We suggest a new working model for glomalose in light of our findings. We hypothesize that glomalose is an abundant polysaccharide specific to mycorrhizal fungi and resides in fungal hyphae and spores (Fig. 7). The specificity of glomalose to mycorrhizal fungi was already suggested by Dr. Wright (Wright *et al*., 1996), and our current data support this hypothesis (Fig. 6). Previous work suggested glomalose as a component of mycorrhizal hyphae and spore cell walls based on localization analysis using immuno‐electron microscopy (Purin & Rillig, 2007). In agreement with findings from Driver *et al*. (2005), we hypothesize that glomalose is used for a physiological function in mycorrhizal fungi and is most likely released to the soil during fungal turnover. Although the actual structure of this cell wall polysaccharide remains to be determined, we hypothesize it is a water‐soluble polysaccharide molecule due to its extraction procedure. Most of the studies on the cell wall of AM fungi focused on intraradical hyphae and arbuscules (Balestrini & Bonfante, 2014; Rich *et al*., 2014) so a more in‐depth characterization of the cell wall from spores and extraradical hyphae will be necessary. Our current working model also fits well with the functional benefits attributed to glomalin/glomalose through years of research (Holátko *et al*., 2021; Irving *et al*., 2021; Fig. 7). Glomalose was named a super‐glue for soils due to its contributions to soil aggregation and aggregate stability (Rillig, 2004; Holátko *et al*., 2021). Soil polysaccharides of bacterial and fungal origin have long been recognized as essential mediators of soil aggregation. Polysaccharides can often serve as a glue and help bind smaller soil particles, leading to aggregation. Soil polysaccharides can provide energy for other filamentous microorganisms supporting the aggregation process. The amount of polysaccharides in soils positively correlates with stable soil aggregates (Martins *et al*., 2012). Particularly, water‐soluble polysaccharides extracted through autoclaving correlate better with aggregate stability than total soil polysaccharides (Puget *et al*., 1998). Hydrophilic polysaccharides help with soil water retention, another vital role of glomalose for soil function (Lowe, 1978). Glomalose was reported to contain a lot of iron (0.04–8.8%), and the fungal cell wall is an important iron store (Rillig *et al*., 2001; Nichols, 2003; Philpott, 2006). Furthermore, polysaccharides contribute substantially to long‐term carbon storage, an essential soil function attributed to glomalose (Rillig *et al*., 2001; Wang *et al*., 2017). The alignment of these previous findings related to soil polysaccharides with the suggested functional role of glomalose further supports our current working model. While the benefits of glomalin/glomalose can yet only be correlated with the abundance of the MAb32B11 antigen rather than proven to originate from it, if the antigen is an abundant cell wall polymer, a causal link is more likely.

## Competing interests

None declared.

## Author contributions

J‐MA, BA, BK and HH designed experiments and interpreted data. DF and DD provided material. BA, BK, LK, CM and HH performed the experiments. J‐MA, BA, BK, HH, DF, LK, CM, TI and DD wrote the manuscript. BA and HH contributed equally to this work.

## Disclaimer

The New Phytologist Foundation remains neutral with regard to jurisdictional claims in maps and in any institutional affiliations.

## Supporting information


**Dataset S1** Raw data from all experiments conducted in this study.

**Fig. S1** Discrepancy between ELISA and Bradford data for glomalin quantification for multiple fungi.
**Fig. S2** Technical improvements of glomalin detection through indirect ELISA.
**Fig. S3** Decrease in glomalin detection by ELISA after air‐drying soil sample.
**Fig. S4** Testing the activity of proteinase K enzyme.
**Fig. S5** Glomalin extraction process extracts carbohydrates.
**Fig. S6** Testing different fungal cell wall polysaccharides as glomalin candidates.
**Fig. S7** Gellan gum is not glomalin.
**Methods S1** STAR protocol for updated ELISA for detection of glomalin using MAb32B11.
**Table S1** Primers used in this study.
**Table S2** Monosaccharide compositions and total carbohydrate by weight of the total dry sample.
Please note: Wiley is not responsible for the content or functionality of any Supporting Information supplied by the authors. Any queries (other than missing material) should be directed to the *New Phytologist* Central Office.

## Data Availability

Raw data used in this work are provided in Dataset S1.
