# Supplementary material for: From glomalin to glomalose: unraveling the molecular identity of the MAb32B11 antigen
Source: New Phytol. 2025 Jun 6;247(5):2328–41. doi: 10.1111/nph.70253 (PMC12329162; doi:10.1111/nph.70253)
Supplement: Supplementary file 2 — Fig. S1 Discrepancy between ELISA and Bradford data for glomalin quantification for multiple fungi. Fig. S2 Technical improvements of glomalin detection through indirect ELISA. Fig. S3 Decrease in glomalin detection by ELISA after air‐drying soil sample. Fig. S4 Testing the activity of proteinase K enzyme. Fig. S5 Glomalin extraction process extracts carbohydrates. Fig. S6 Testing different fungal cell wall polysaccharides as glomalin candidates. Fig. S7 Gellan gum is not glomalin. Methods S1 STAR protocol for updated ELISA for detection of glomalin using MAb32B11. Table S1 Primers used in this study. Table S2 Monosaccharide compositions and total carbohydrate by weight of the total dry sample. Please note: Wiley is not responsible for the content or functionality of any Supporting Information supplied by the authors. Any queries (other than missing material) should be directed to the New Phytologist Central Office. [file NPH-247-2328-s002.pdf]

New Phytologist **Supporting Information**

Article title: From Glomalin to Glomalose: Unraveling the Molecular Identity of the MAb32B11

Authors: Burcu Alptekin, Hayley Hirsch1, Bailey Kleven, Lauren King, Caitlin McLimans, Dierdra Daniels, Thomas Irving, Daniela Floss, Jean-Michel Ané

Article acceptance date: 8 May 2025

The following Supporting Information is available for this article:

Fig. S1. Discrepancy between ELISA and Bradford data for glomalin quantification for multiple fungi.

Fig. S2. Technical improvements of glomalin detection through indirect ELISA.

Fig. S3. Decrease in glomalin detection by ELISA after air drying soil sample.

Fig. S4. Testing the activity of Proteinase K enzyme.

Fig. S5. Glomalin extraction process extracts carbohydrates.

Fig. S6. Testing different fungal cell wall polysaccharides as glomalin candidates.

Fig. S7. Gellan gum is not glomalin.

Table S1. Primers used in this study

Table S2. Monosaccharide compositions and total carbohydrate by weight of the total dry sample.

Method S1. STAR protocol for updated ELISA for detection of glomalin using MAb32B11.

Data S1. Raw data from all experiments conducted in this study.

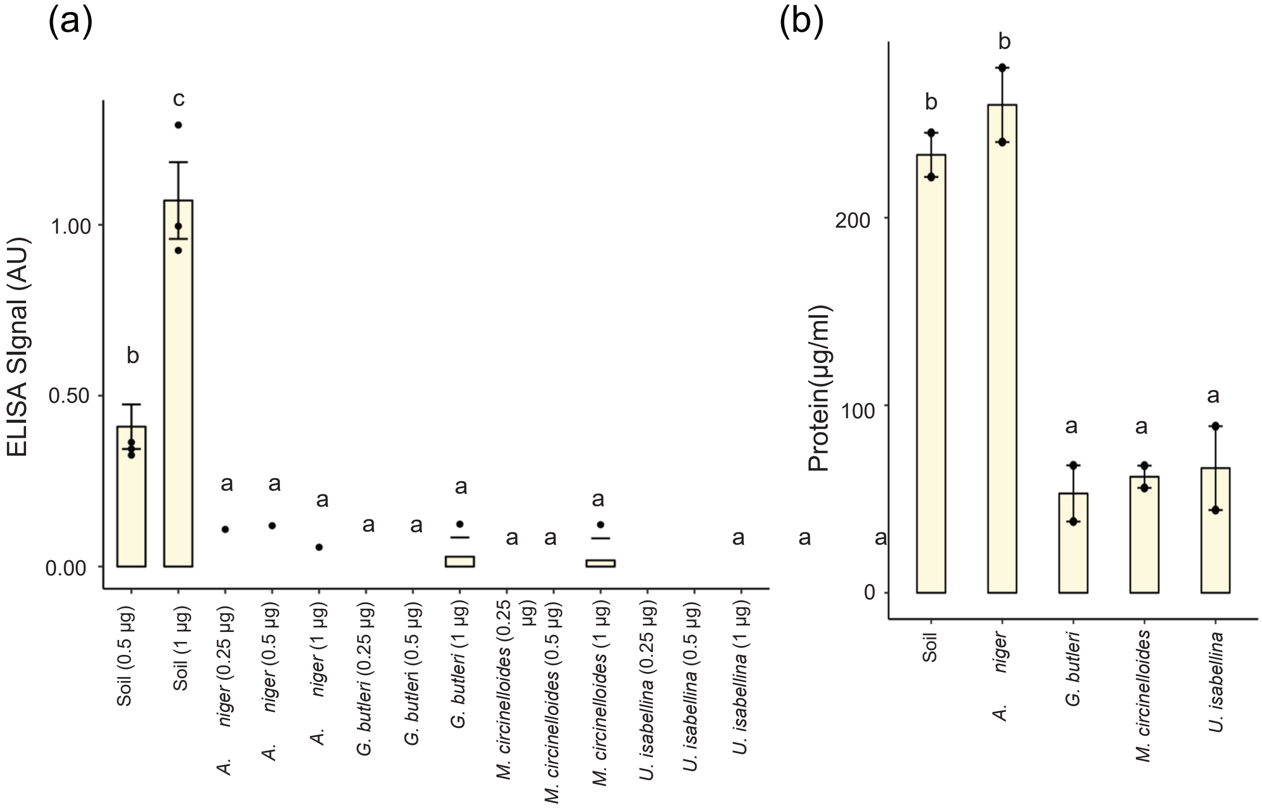

Fig. S1. Discrepancy between ELISA and Bradford data for glomalin quantification for multiple fungi.

(a) Glomalin signal in Arbitrary Units (AU) based on indirect ELISA with MAb32B11 using soil, hyphae/spore samples of a fungus from Ascomycota (*Aspergillus niger*), hyphae/spore samples of a fungus from zygomycota (*Gongronella butleri*), and hyphae/spore samples of two fungi from Mucoromycota (*Mucor circinelloides* and *Umbelopsis isabellina*). Soil samples show an increased amount of glomalin signal in response to an increased amount of antigen, which was provided by increasing the amount of soil proteins; however, different fungal samples did not show this signal increase (One-Way ANOVA, n = 4, p <0.05) **(b)** Bradford reactive proteins soil, hyphae/spore samples of a fungus from Ascomycota (*Aspergillus niger)*, hyphae/spore samples of a fungus from zygomycota (*Gongronella butleri*), and hyphae/spore samples of two fungi from Mucoromycota (*Mucor circinelloides* and *Umbelopsis isabellina*). Statistical significances are based on one-way ANOVA post-hoc Tukey's HSD test (n = 2, p <0.05). Error bars represent standard error of the mean.

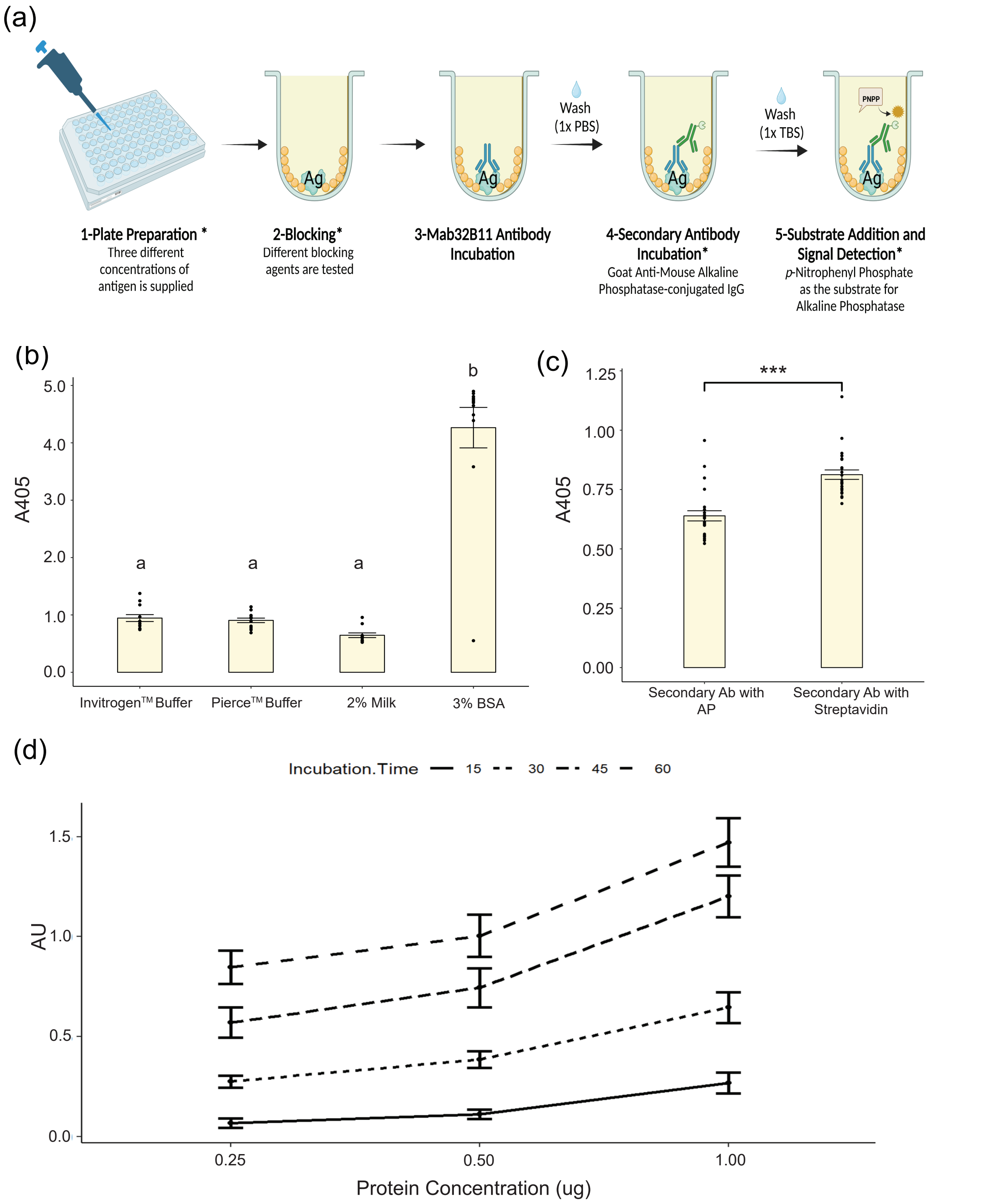

Fig. S2. Technical improvements of glomalin detection through indirect ELISA. (a) Different steps of indirect ELISA method for glomalin detection is illustrated. Multiple changes to the original protocol (Wright *et* al., 1996) are suggested covering several steps of indirect ELISA (marked with *). **(b)** Different blocking buffers were tested to decrease the ELISA background level. The change in the blank signal in response to the use of different blocking buffers is represented (one-way ANOVA, $n = 12$, $p < 0.05$). **(c)** The secondary antibody used in the original protocol, anti-mouse IgM labeled with biotin, was replaced by an anti-mouse IgG labeled with alkaline phosphatase. The change in blank signal with the use of the new Goat Anti-Mouse Alkaline Phosphatase-conjugated IgG is shown (Student's t-test, n=12, p <0.05: *, p<0.01 **, p<0.001 ***). **(d)** The incubation time for p-nitrophenyl phosphate (PNPP) is suggested as at least 45 minutes to get a better resolution of the ELISA signal. The graph shows the signal difference in Arbitrary Units (AU) after 15, 30, 45 and 60 minutes of incubation with PNPP at the end of indirect ELISA. Error bars represent the standard error of 12 technical replicates for each concentration.

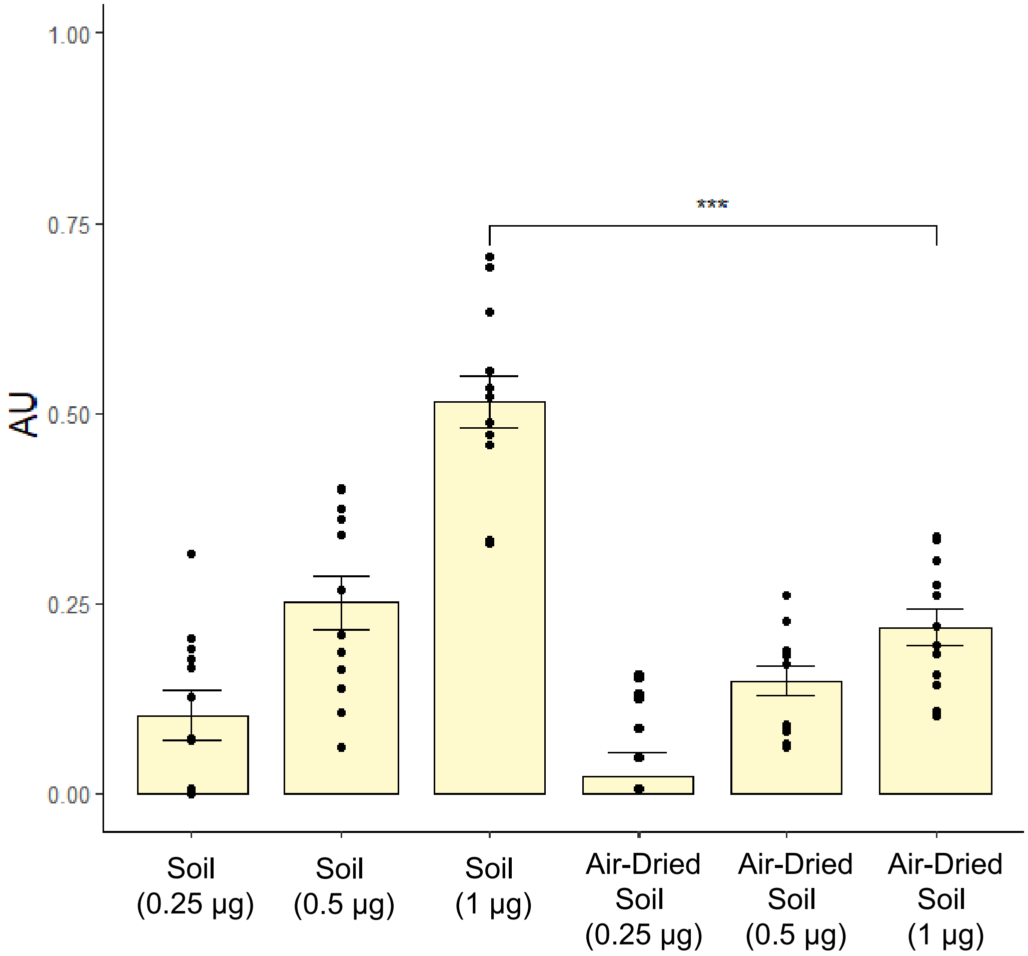

Fig. S3 Decrease in glomalin detection by ELISA after air drying soil sample. Measuring glomalin signal from non air-dried soil (Soil) and soil air-dried for 30 days (Air-Dried Soil) in arbitrary units with indirect ELISA. Soil and Air-Dried Soil were added to ELISA plates in increasing protein concentration (0.25 µg, 0.5 µg and 1 µg). Dots represent technical replicates (n = 12) and bars represent mean ELISA signal + SEM (Kruskall-Wallis with post hoc Pairwise Wilcoxon Rank Sum test, n = 12; *, $P \leq 0.05$; **, $P \leq 0.01$; ***, $P \leq 0.001$.).

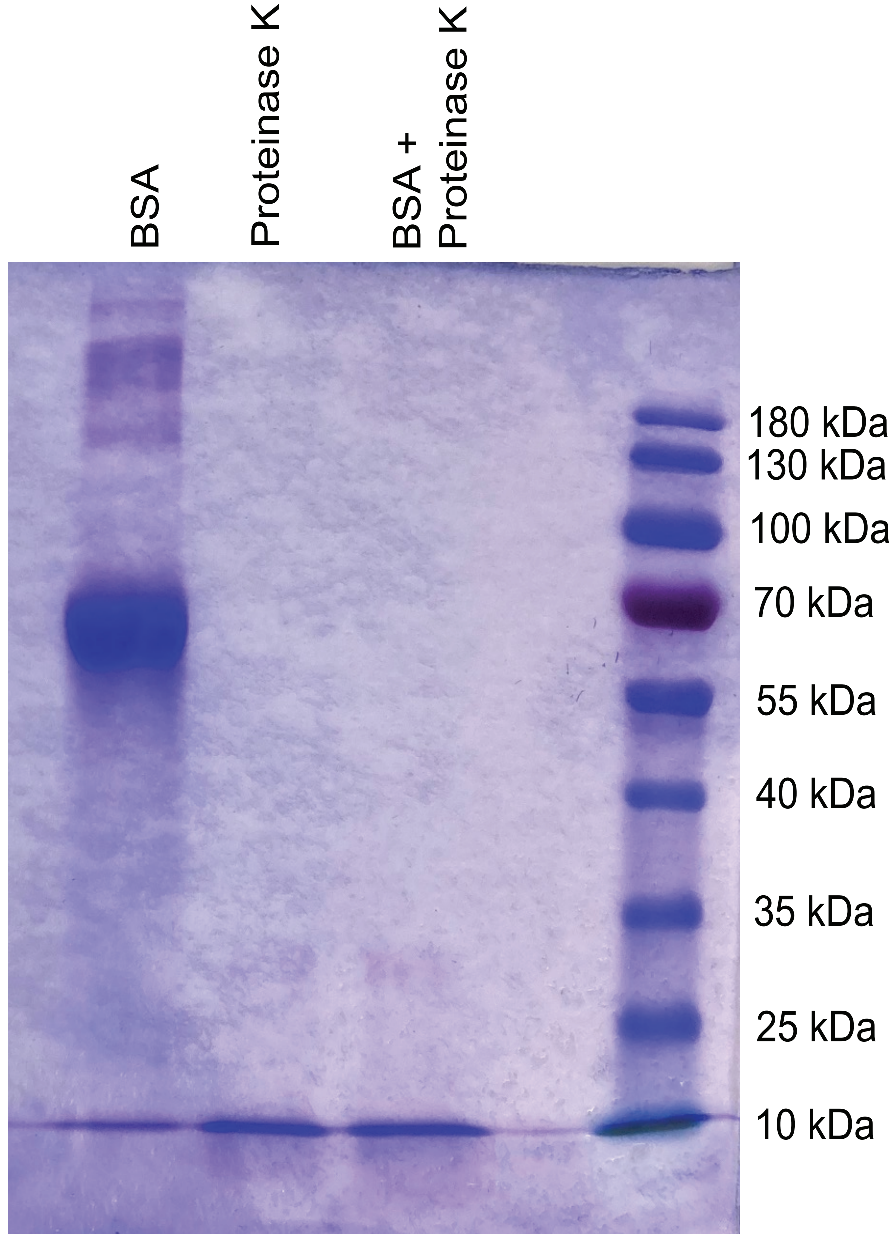

Fig. S4 Testing the activity of Proteinase K enzyme. Prior to treatment of glomalin extracts with Proteinase K enzyme, the enzymatic activity of Proteinase K was confirmed using Bovine Serume Albumin (BSA) as a control. BSA (5 µg) was treated with 10 µg of Proteinase K and incubated for 8 hours at 37 C. As shown with SDS-PAGE, BSA (66 kDa) treated with Proteinase K is degreded completely.

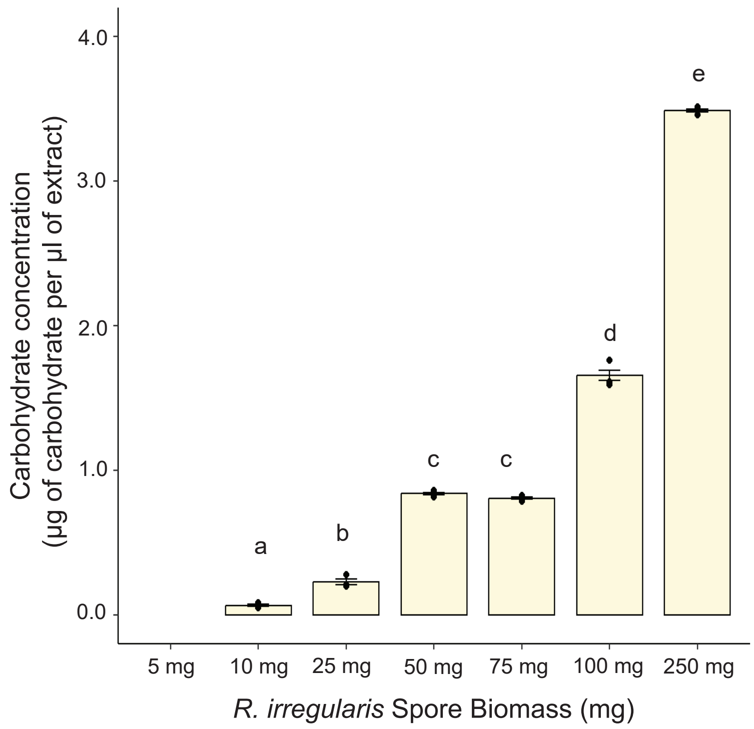

Fig. S5. Glomalin extraction process is extracting carbohydrates. Increase in carbohydrate concentration per µl of glomalin extract in response to the increase in *R. irregularis* spore biomass is shown. The increased concentration of *R. irregularis* spore biomass used in the extraction leads to a distinct increase in carbohydrate concentration; no protein was detected in any of the extracts from spore samples (one-way ANOVA with post hoc Tukey's HSD test, n= 2, $p<0.05$). Error bars represent standard error of the mean.

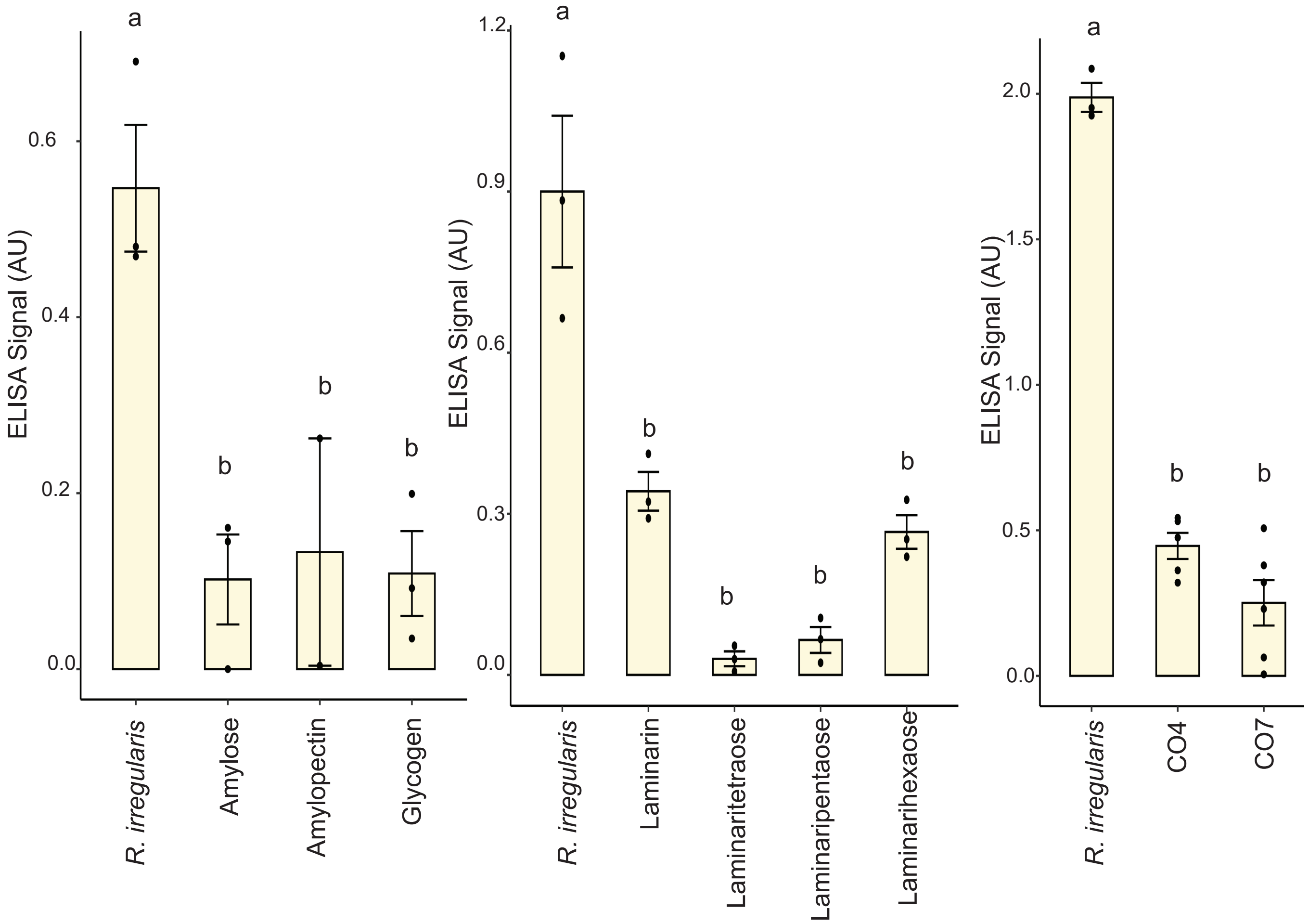

Fig. S6. Testing different fungal cell wall polysaccharides as glomalin candidates. The cross-reactivity of various fungal cell wall polysaccharides with MAb32B11 was tested using 5 and 10 µg of corresponding carbohydrates. Glomalin extract from *R. irregularis* hyphae and spore samples was used as a positive control in the ELISA. Statistical analysis of the samples were done with one-way ANOVA with post hoc Tukey's HSD test, $p < 0.05$). Error bars in the graph represent standard error of the mean.

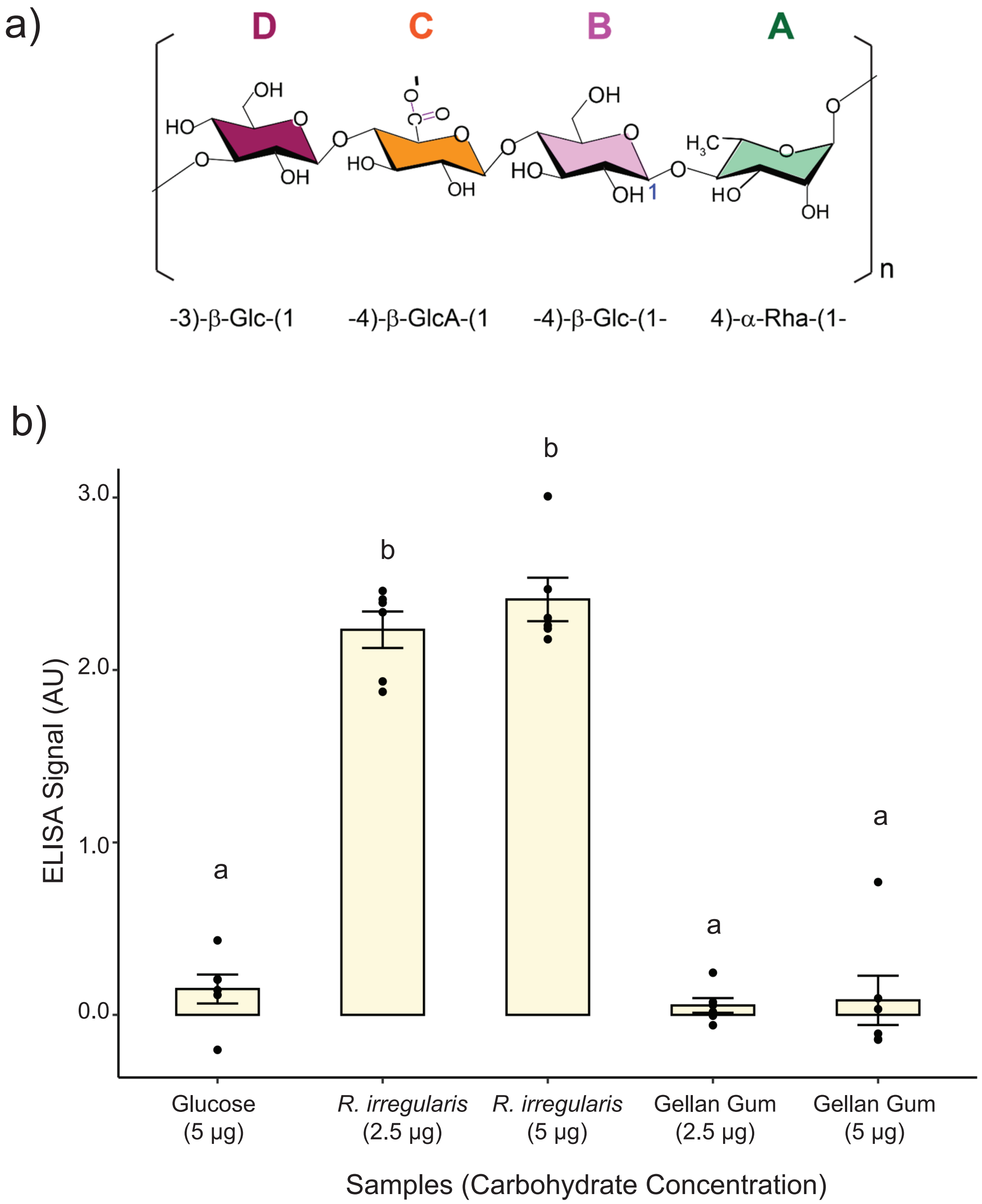

Fig. S7. Gellan gum is not glomalin. 2D NMR analysis, combined with GC-MS results predicted the structure of the polysaccharide present in Fraction 2 as represented in **(a)**. This structure is the same as gellan gum produced by Sphingomonas elodea. The cross reactivity of Gellan gum with MAb32B11 antibody was tested using ELISA **(b)**. Gellan gum was prepared as 5 µg/L in water and autoclaved following a similar protocol to glomalin extraction. At two tested concentrations 2.5 and 5 µg of carbohydrates per well, gellan gum did not show cross reactivity with MAb32B11 antibody, therefore, it is most likely not glomalin (one-way ANOVA with post hoc Tukey's HSD test, $p < 0.05$). Error bars represent standard error of the mean.

Table S1. Primers used in this study

Primers for generating protein construct	
RiHSP60_F_TOPO	CAC CAT GCA GCG TGT CAG TCA ATT
RiHSP60_R	TTA CAT CAT GCC CAT GTC TCC

Table S2. Monosaccharide compositions and total carbohydrate by weight of the total dry sample.

Glycosyl Residues	Fraction 2	
	Mass (µg)	Mol %
Arabinose (Ara)	0.03	1.1
Rhamnose (Rha)	1.5	48.4
Fucose (Fuc)	0.02	0.6
Glucose (Glc)	1.4	41.2
Mannose (Man)	0.82	0.9
Galactose (Gal)	0.1	1.9
Glucoronic acid (GlcA)	0.03	3.1
N-acetyl galactosamine (GalNAc)	0.1	2.8
Sum	3.2	100
Total carbohydrate by weight %	~3%	-

Method S1. STAR protocol for updated ELISA for detection of glomalin using MAb32B11.

Indirect ELISA protocol for the detection of glomalin in hot-citrate extracts from soil

Burcu Alptekin[1,3,*], Hayley Hirsch[1], Bailey Kleven[2], Jean-Michel Ané[1,4,**]

[1]Bacteriology Department, University of Wisconsin – Madison, Madison, WI, 53706, USA

[2]Biochemistry and Molecular Biology Department, Michigan State University, East Lansing, MI, 48824, USA

[3]Technical contact

[4]Lead contact

*Correspondence: alptekin@wisc.edu

**Correspondence: jeanmichel.ane@wisc.edu

Summary

In 1996, Dr. Wright developed an indirect ELISA protocol to detect the presence of a beneficial soil substance, glomalin [1]. Our modified protocol relies on detecting glomalin based on total carbohydrate content in samples. We added a positive control (three concentrations of glomalin extract from soil) and an alternative secondary antibody to improve the reliability of glomalin detection in samples. Additionally, we use the same blocking agent as proven superior in our experiments.

For complete details on the use and execution of this protocol, please refer to Wright et al.[1]

Before you begin

The protocol below describes the specific steps for detecting glomalin in hot-citrate extracts from soil samples. However, we have also used this protocol for hot-citrate extracts from fungi, including R*hizophagus irregularis* spores and hyphae.

<u>*Perform Glomalin Extraction*</u> **(Timing: 2 h)**

1. Conduct easily-extractable glomalin extraction on soil samples as previously described by Wright and Upadhyaya[2].

Note: We recommend not drying the soil before conducting glomalin extraction.

a. Homogenize soil samples via sieving and grinding

Note: We used a 500 µm sieve and a standard blender for our experiments.

b. Using a standard scale, measure 1 g of homogenized soil sample and place it in a 50 mL Falcon tube.
c. Mix 1 g of soil with 8 mL of 20 mM sodium citrate buffer pH 7.0.
d. Mix the sample thoroughly with vortexing before caping it with aluminum foil.
e. Autoclave sample for 30 minutes at 121℃.
f. Immediately after autoclaving, centrifuge samples at 3,000 x g for 15 minutes
g. Within a sterile environment, remove the supernatant from the pellet and place it in a sterile 50 mL Falcon tube.

Note: The size of the storage test tube does not matter, but it must be sterile to prevent contamination.

h. Store supernatant at 4℃ for further analysis.

Note: We recommend storing samples for no more than 2 months.

Measure Total Carbohydrate Concentration **(Timing: 1 h)**

2. Quantify the total carbohydrate concentration in glomalin extract using phenol-sulfuric acid method [3].

Note: We used the Total Carbohydrate Assay Kit from Sigma-Aldrich (Catalog # MAK104, Manufacturer's Protocol). The kit recommends measuring the absorbance at 490 nm. We used the Fisherbrand™ accuSkan™ FC Filter-Based Microplate Photometer (Catalog # 14-377-575), and it does not have the capability to read absorbance at 490 nm, so we measured the absorbance at 450 nm [3].

Key resources table

REAGENT or RESOURCE	SOURCE	IDENTIFIER
Antibodies		
Mouse monoclonal anti-glomalin (MAb32B11)	Wright et al.[1]	NA
Goat polyclonal anti-mouse IgG	Sigma-Aldrich	Cat#A9316
Chemicals, peptides, and recombinant proteins		
1-Step™ PNPP Substrate Solution	Thermo Fisher	Cat#37621
Software and algorithms		
RStudio	This paper	http://www.rstudio .com/
Other		
25 mL Graduated Reservoir, White	USA Scientific,	Cat#2330-2235
Wellwash™ Microplate Washer	Thermo Fisher	Cat#5165000

Materials and equipment

Phosphate Buffered Saline (1X PBS) pH 7.4

Reagent	Final concentration	Amount
NaCl	137 mM	16.01 g
KCl	2.7 mM	0.4 g
Na_2HPO_4	10 mM	2.84 g
K_2HPO_4	2 mM	0.7 g
ddH_2O	n/a	2L
**Total**	**n/a**	**2 L**

Storage conditions: Stored at room temperature for up to 2 months.

Phosphate Buffered Saline with Tween 20 (1X PBS-T) pH 7.4

**Reagent**	**Final concentration**	**Amount**
NaCl	137 mM	16.01 g
KCl	2.7 mM	0.4 g
Na_2HPO_4	10 mM	2.84 g
K_2HPO_4	2 mM	0.7 g
Tween 20	0.02%	0.4 mL
ddH_2O	n/a	1.96 mL
**Total**	**n/a**	**2 L**

Storage conditions: Stored at room temperature for up to 2 months.

- Tris Buffered Saline (1X TBS) pH 7.4: dissolve 29.22 g of NaCl (250 mM) and 2.42 g (10 mM) Tris Base into 2 L ddH_2O.

Tris Buffered Saline with Tween 20 (1X TBS-T) pH 7.4

**Reagent**	**Final concentration**	**Amount**
NaCl	250 mM	29.22 g
Tris Base	10 mM	2.42 g
Tween 20	0.02%	0.4 mL
ddH_2O	n/a	1.96 mL
**Total**	**n/a**	**2 L**

Storage conditions: Stored at room temperature for up to 2 months.

- D-(+)-Glucose Solution (2 mg/mL): dissolve 20 mg of D-(+)-Glucose in 10 mL ddH_2O. Filter sterilize with 0.22 µm PVDF syringe filter. Stored at 4°C for 1 month.
- Blocking Buffer (2% (w/v) non-fat milk in 1X PBS pH 7.4): dissolve 1 g of non-fat milk powder in 50 mL of 1X PBS pH 7.4. Made fresh before each assay.
- Primary Antibody Solution: dilute MAb32B11 1:500 in 1X PBS pH 7.4. Made fresh before each assay

- Secondary Antibody Solution: dissolve 0.1 g of bovine serum albumin (BSA) (1%) in 10 mL 1X PBS pH 7.4 with gentle inversion. Dilute anti-Mouse IgG 1:1000 in 1X PBS pH 7.4 with 1% (w/v) BSA. Made fresh before each assay.

Alternatives: All wash steps of the ELISA protocol can be performed by hand with rinsing over the sink and tapping out plates thoroughly on a paper towel. An automated ELISA well washer is not necessary, but it is convenient. We used Thermo Scientific™ 96-well Microtiter™ Microplates (Thermo Fisher Scientific, Cat#14-245-73).

Alternatives: If processing a large amount of ELISA plates (>4 plates), we recommend adding stop solution to wells after final incubation with the substrate (1-Step™ PNPP Substrate Solution). We used PNPP Stop Solution (Leinco Technologies, Product # P227).

Step-by-step method details

<u>*Make ELISA plate*</u> **(Timing: 1 day)**

Up to one day before running the ELISA, the ELISA plate is designed and generated after samples are added to wells based on the total carbohydrate content.

1. Generate a completely randomized ELISA plate design
    a. The plate must contain the following samples: blank (1X PBS), negative control (D-(+)-Glucose Solution), and positive control (soil with detectable GRSP/IRSP content).
    b. All samples with a detectable carbohydrate content must be added in at least three different concentrations. We use 0.05 µg, 0.5 µg, and 5 µg of carbohydrate per well.
    c. All samples must have AT LEAST three technical replicates
2. Calculate the volumes of sample needed to place in each well based on total carbohydrate concentration (µg of carbohydrate per µL of glomalin extract).

Note: Each well, regardless of the total µg of carbohydrate in the well, must contain a final volume of 50 µL.

a. Divide the desired carbohydrate concentration per well (ex: 0.5 µg per well) by the total carbohydrate concentration (µg of carbohydrate/µL of extract).
    i. This value equals the microliters of sample extract needed to reach the desired µg of carbohydrate per well.
b. Subtract the microliters of sample extract needed to reach the desired well concentration (i.e., µg of carbohydrate per well) from 50 µL.
    i. This value is the microliters of 1X PBS to add to the well to reach the total volume (50 µl)
c. To facilitate plate making, the samples can be pre-diluted in 1X PBS rather than pipetting each sample individually.
    i. To do this, multiply both volume values (i.e., microliters of sample extract needed to reach the desired µg of carbohydrate per well and microliters of 1X PBS to add to the well for a total volume (50 µL) by the number of wells desired (ex: If I need enough sample for 10 technical replicates, I multiply both values by 10)
        1. This calculation produces
            a. microliters of sample needed to reach the desired µg of carbohydrate for X technical replicates
            b. microliters of 1X PBS needed to reach total volume (50 µL) for X technical replicates
        2. Combine these volumes in a test tube and then pipette 50 µL of pre-diluted sample into each well

Note: Always plan for more technical replicates than needed to avoid running out of samples due to pipette error.

3. Pipette 50 µl of each sample into ELISA wells based on design.
4. Allow the samples to bind to wells overnight at room temperature.

Note: The ELISA plate may not be dry overnight, so more time may be needed.

CRITICAL: Do not run ELISA until the samples have dried in wells otherwise, the sample will wash out of the wells as the assay progresses.

Run ELISA **(Timing: 3 h 30 min)**

ELISA protocol is conducted to detect the presence of glomalin in hot-citrate extract based on the ELISA signal when comparing samples and concentrations.

Optional: Before starting, create and save a program on the Wellwash™ Microplate Washer (Thermo Fisher, Catalog # 5165000, Manual) that includes 3 wash cycles. Additionally, ensure the Buffer A chamber is filled with 1X PBS-T and the system is primed.

5. Add 250 µL of freshly prepared blocking buffer to each well.

Note: Blocking buffer can be poured into a reservoir for easier access with a multi-channel pipette.

6. Incubate the ELISA plate on an orbital shaker (1 rcf) for 15 minutes at room temperature.
7. Invert the plate over a sink, followed by blotting by vigorously tapping the inverted plate on an absorbent paper towel.
8. Add 50 µL of the primary antibody solution to each well.
9. Incubate the ELISA plate on the orbital shaker (1 rcf) for 1 hour at room temperature.
10. Invert the plate over a sink, followed by blotting by vigorously tapping the inverted plate on an absorbent paper towel.
11. Wash the ELISA plate 3 times with 1X PBS-T pH 7.4
    a. Pour 1X PBS-T pH 7.4 over the plate, then invert the plate to the sink.
    b. Repeat step 11a three times.
    c. After three washes, tap the inverted plate vigorously onto the countertop covered in an absorbent paper towel.

Optional: Use the Wellwash™ Microplate Washer to wash the plates with 1X PBS-T pH 7.4 three times. After use, rinse the Buffer A chamber three times with ddH_2O, fill the chamber with 1X TBS-T pH 7.4, and prime the machine.

12. Add 50 µL of secondary antibody solution to each well.
13. Incubate the ELISA plate on the orbital shaker (1 rcf) for 1 hour at room temperature.
    a. At the start of the secondary antibody incubation, remove 1-Step™ PNPP Substrate Solution from 4°C to equilibrate to room temperature.
14. Invert the plate over a sink, followed by blotting by vigorously tapping the inverted plate on an absorbent paper towel.
15. Wash the ELISA plate 3 times with 1X TBS-T pH 7.4
    a. Pour 1X TBS-T pH 7.4 over the plate then invert the plate over the sink.
    b. Repeat step 15a three times.
    c. After three washes, tap the inverted plate vigorously onto the countertop covered in an absorbent paper towel.

Optional: Use the Wellwash™ Microplate Washer to wash the plates with 1X TBS-T pH 7.4 three times. After use, rinse out the Buffer A chamber three times with ddH_2O.

16. Add 100 µL of 1-Step™ PNPP Substrate Solution to each well.
17. Cover the ELISA plate with a sheet of aluminum foil.
18. Incubate the ELISA plate on the shaker (1 rcf) at room temperature for 45 minutes. Measure the absorbance at 405 nm (A_{405}) with the plate reader at the 30-minute and 45-minute time points.

Optional: If processing multiple plates, add PNPP Stop Solution (Leinco Technologies, Product # P227, Manufacturer's Protocol) to wells at the 45-minute time point.

Expected outcomes

The expected outcome is the specific detection of glomalin within the soil glomalin extract based on absorbance at 405 nm (A_{405}) above the background signal and positively correlated with carbohydrate concentration. Therefore, A_{405} from the blank (1X PBS pH 7.4) and negative control (D-(+)-Glucose Solution) should be equal. The positive control (soil glomalin extract) should have a higher A_{405} than the blank and negative control. Additionally, as carbohydrate concentration increases in wells, A_{405} should increase in the positive control but should not change in the blank or negative control.

Quantification and statistical analysis

1. Calculate ELISA signal in arbitrary units (AU) for positive and negative control samples.
    a. Calculate the average absorbance at 405 nm (A_{405}) for the blank (1X PBS) samples
    b. Convert the absorbance data for each sample into arbitrary units (AU)
        i. Calculate the adjusted absorbance (A_{405}) value of each technical replicate.
            1. Adjusted absorbance value = absorbance (A_{405}) value of individual technical replicate – average absorbance (A_{405}) of the blank (1X PBS)
        ii. Calculate the arbitrary unit assigned to each sample.
            1. Arbitrary unit (AU) = the average of the adjusted absorbance for the sample
        iii. Calculate the standard error of the adjusted absorbance value for each sample.
        iv. With the adjusted absorbance values, apply the Shapiro-Wilk test across the data set to assess normality.
        v. Based on normality, run either:
            1. ANOVA or Kruskal-Wallis for multiple comparisons
            2. Student`s T-test or Wilcoxon Test for comparing two groups

Limitations

As glomalin content varies across soils with geographic location, soil properties, and biodiversity, the difference in ELISA signal (AU) between the soil glomalin extract with the blank and negative control will vary [2,4].

Resource availability

Lead contact: Dr. Jean-Michel Ané can be reached at jeanmichel.ane@wisc.edu

Materials availability: All the information regarding the materials is provided in the above protocol

Data and code availability: Not available

Acknowledgments

Valent BioSciences, a subsidiary of Sumitomo Chemical Co., provided funding for this research to J.M.A. The authors thank Arlen Slaymaker and Dean Yun for technical assistance. The authors also thank Dr. Thomas Barrick Irving and Oswaldo Valdes-Lopez for their valuable discussions and insightful comments on the project.

Author contributions

JMA, BA, BK, and HHH designed experiments and interpreted data. BA, BK, and HHH performed the experiments. JMA, BA, HHH, and BK wrote the protocol.

Declaration of interests

The authors declare that they have no conflict of interest.

References

1. Wright, S.F., Franke-Snyder, M., Morton, J.B., and Upadhyaya, A. (1996). Time-course study and partial characterization of a protein on hyphae of arbuscular mycorrhizal fungi during active colonization of roots. Plant Soil *181*, 193–203.

2. Wright, S.F., and Upadhyaya, A. (1998). A survey of soils for aggregate stability and glomalin, a glycoprotein produced by hyphae of arbuscular mycorrhizal fungi. Plant Soil *198*, 97–107.

3. Nielsen, S.S. (2017). Total Carbohydrate by Phenol-Sulfuric Acid Method. In Food Analysis Laboratory Manual, S. S. Nielsen, ed. (Springer International Publishing), pp. 137–141.

4. Lovelock, C.E., Wright, S.F., Clark, D.A., and Ruess, R.W. (2004). Soil Stocks of Glomalin Produced by Arbuscular Mycorrhizal Fungi across a Tropical Rain Forest Landscape. J. Ecol. *92*, 278–287.
